# Conformational Dynamics and Binding Free Energies of Inhibitors of BACE-1: From the Perspective of Protonation Equilibria

**DOI:** 10.1371/journal.pcbi.1004341

**Published:** 2015-10-27

**Authors:** M. Olivia Kim, Patrick G. Blachly, J. Andrew McCammon

**Affiliations:** 1 Department of Chemistry and Biochemistry, University of California San Diego, La Jolla, California, United States of America; 2 Department of Pharmacology, University of California San Diego, La Jolla, California, United States of America; 3 Howard Hughes Medical Institute, University of California San Diego, La Jolla, California, United States of America; 4 National Biomedical Computation Resource, University of California San Diego, La Jolla, California, United States of America; UNC Charlotte, UNITED STATES

## Abstract

BACE-1 is the β-secretase responsible for the initial amyloidogenesis in Alzheimer’s disease, catalyzing hydrolytic cleavage of substrate in a pH-sensitive manner. The catalytic mechanism of BACE-1 requires water-mediated proton transfer from aspartyl dyad to the substrate, as well as structural flexibility in the flap region. Thus, the coupling of protonation and conformational equilibria is essential to a full *in silico* characterization of BACE-1. In this work, we perform constant pH replica exchange molecular dynamics simulations on both apo BACE-1 and five BACE-1-inhibitor complexes to examine the effect of pH on dynamics and inhibitor binding properties of BACE-1. In our simulations, we find that solution pH controls the conformational flexibility of apo BACE-1, whereas bound inhibitors largely limit the motions of the holo enzyme at all levels of pH. The microscopic pK_a_ values of titratable residues in BACE-1 including its aspartyl dyad are computed and compared between apo and inhibitor-bound states. Changes in protonation between the apo and holo forms suggest a thermodynamic linkage between binding of inhibitors and protons localized at the dyad. Utilizing our recently developed computational protocol applying the binding polynomial formalism to the constant pH molecular dynamics (CpHMD) framework, we are able to obtain the pH-dependent binding free energy profiles for various BACE-1-inhibitor complexes. Our results highlight the importance of correctly addressing the binding-induced protonation changes in protein-ligand systems where binding accompanies a net proton transfer. This work comprises the first application of our CpHMD-based free energy computational method to protein-ligand complexes and illustrates the value of CpHMD as an all-purpose tool for obtaining pH-dependent dynamics and binding free energies of biological systems.

## Author Summary

Formation of insoluble amyloid plaques in the vascular and hippocampal areas of the brain characterizes Alzheimer’s disease, a devastating neurodegenerative disorder causing dementia. Site-specific hydrolytic catalysis of β-secretase, or BACE-1, is responsible for production of oligomerative amyloid β-peptide. As the catalytic activity of BACE-1 is pH-dependent and its structural dynamics are intrinsic to the catalysis, we examine the dependence of dynamics of BACE-1 on solution pH and its implications on the catalytic mechanism of BACE-1. Also, we highlight the importance of accurate description of protonation states of the titratable groups in computer-aided drug discovery targeting BACE-1. We hope the understanding of pH dependence of the dynamics and inhibitor binding properties of BACE-1 will aid the structure-based inhibitor design efforts against Alzheimer’s disease.

## Introduction

Alzheimer’s disease is a neurodegenerative disorder characterized by loss of memory and failure in cognitive abilities, resulting from synaptic dysfunction and neuronal death in the brain [1–5]. Major damages found in the brains of Alzheimer’s patients include cerebral and vascular deposits of insoluble amyloid plaques, consisting of aggregates of amyloid β-peptide (Aβ) [6–8]. Aβ occurs in two different forms, Aβ_40_ and Aβ_42_, and the overproduction and oligomerization of Aβ_42_ is associated with the early onset of Alzheimer’s disease [9–12]. Aβ is produced by sequential proteolytic cleavage of the type 1 transmembrane protein amyloid precursor protein (APP) by β- and γ-secretases [13,14]. While γ-secretase generates several Aβ peptides varying in the length of C-termini, β-secretase, or β-site APP cleaving enzyme 1 (BACE-1), cleavage precisely gives the fibrillogenic Aβ_42_ [13–15]. Therefore, as it catalyzes the initial site-specific hydrolysis step of Aβ production, BACE-1 is an attractive therapeutic target for the treatment of Alzheimer’s disease [1–3,16,17].

**Fig 1:**
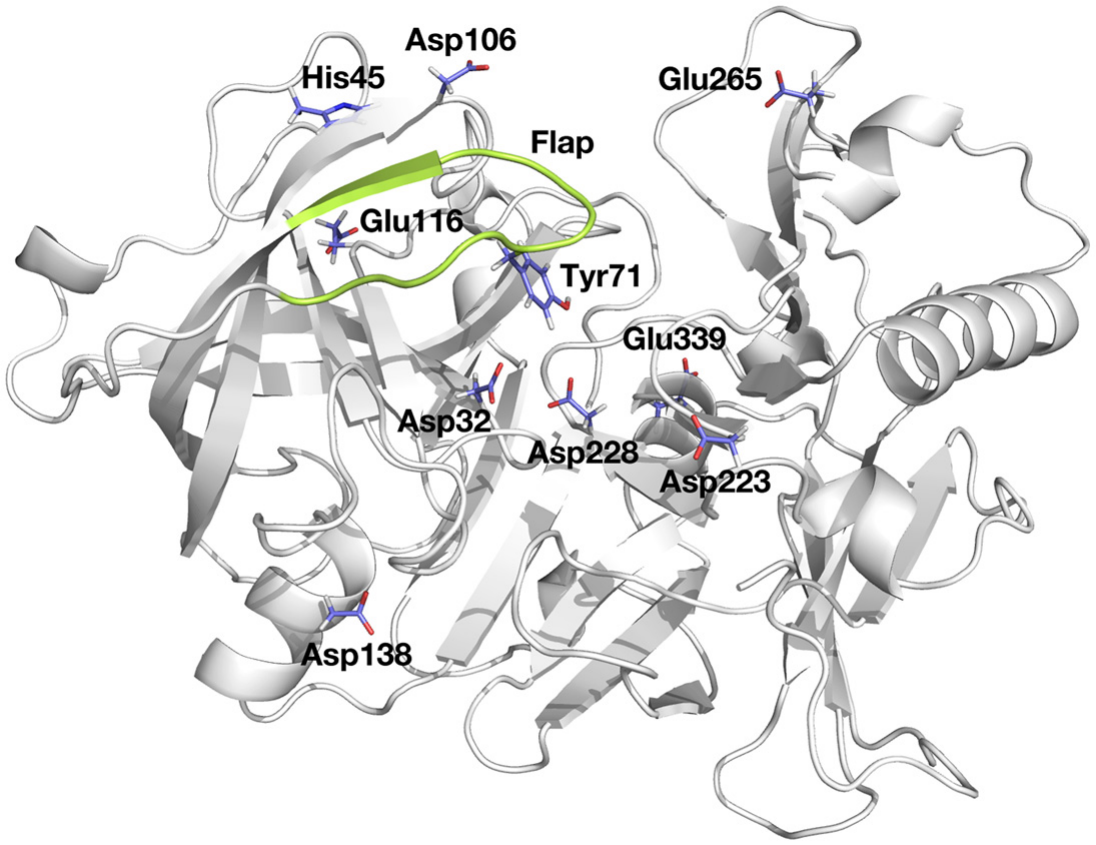
Structure of BACE-1, highlighted with titratable residues considered here and flap region (residues 67 to 77) in green.

As an aspartyl protease, the catalytic mechanism of BACE-1 involves two highly conserved aspartyl residues, Asp32 and Asp228, which form a symmetric dyad at the base of the catalytic cleft of the enzyme (Fig 1) [16]. Analogous aspartyl dyads are found in the aspartyl protease family including pepsin, cathepsin D, renin, and HIV-1 protease [18–21]. The dyad is central to the hydrolytic cleavage of the substrate through a nucleophilic attack of water bound to the dyad [19–23]. Due to the general acid-base catalytic nature of the mechanism, the enzymatic activity of BACE-1 is maximal at pH 4.5 and strongly depends on solution pH [24,25].

The active site of BACE-1 is covered by an antiparallel β hairpin (henceforth referred to as the flap region; residues 67 to 77 shown in green in Fig 1) that is characteristic of aspartyl proteases [16,26–29]. The X-ray crystal structures of other aspartyl proteases indicate that the flap is inherently flexible [26–29]. The flexibility of the flap region is likely utilized in catalysis, with transitions between open and closed conformations facilitating the entrance of substrates into the active site and release of hydrolytic products [21,29–31]. The conserved Tyr71 [20] located at the tip of the flap region is particularly essential for the conformational transitions of the flap. Observations from X-ray crystallographic structures and molecular dynamics (MD) simulations suggest that variation in hydrogen bond patterns between Tyr71 and surrounding residues such as Lys107, Lys75, Gly74, Glu77, and Trp76 enables the flexible motions of the flap [21,29,31–33]. In the presence of inhibitors, Tyr71 can directly interact with bound inhibitors and lock the flap in the closed state [31,33,34].

Given that the enzymatic activity of BACE-1 depends on solution pH and that the structural flexibility is intrinsic to catalysis, a comprehensive understanding of the pH dependence of BACE-1 dynamics would greatly benefit drug design efforts. A detailed description of the protonation state of the aspartyl dyad is also important as all known bound inhibitors directly contact the dyad. Several computational efforts have attempted to determine the protonation state of the dyad, employing methods such as molecular mechanics (MM) [35], quantum mechanics (QM) [36], QM/MM [37], molecular docking [38,39], and continuum electrostatics calculations [40]. However, the conformational flexibility of BACE-1 was not rigorously addressed in these computations.

The importance of accounting for conformational flexibility in pK_a_ computations has been well established [41–46]. The instantaneous pK_a_ of a titratable group is determined by its electrostatic environment, which is affected by the given conformation of protein and protonation states of other titratable residues [47]. Changes to the conformation of the protein can alter the electrostatics, which may, in turn, induce a shift in the pK_a_ of titratable groups. The prevalence of such coupling of protonation and conformational equilibria has been observed in various systems both computationally and experimentally [44,48–59]. Furthermore, complex formation between protein and small molecules or other proteins can also induce changes in the pK_a_ values of titratable groups on either binding partner [51,60–70].

Consequently, several computational methods have been developed to explicitly account for conformational changes in pK_a_ computations [49,71–76]. Among these, various flavors of constant pH molecular dynamics (CpHMD) methodologies have emerged to incorporate pH as an additional external thermodynamic variable to the conventional MD framework [48,77–81]. CpHMD simulations have been successfully applied to predict pK_a_ values of titratable groups in proteins [48,77–83] and nucleic acids [84–86], as well as to explain the acid-base catalysis by RNase A [87] and to understand the mechanisms behind the pH-dependent conformational changes [59,88].

Conventional molecular simulations or free energy computations typically employ fixed protonation states that are identical for both free and bound states, set prior to the computations. This assumption ignores the possibility of protonation states changing upon binding and can lead to significant errors when protein-ligand binding is a pH-dependent process [60]. Furthermore, the pH-dependent conformational dynamics cannot be appropriately addressed if the protonation states are fixed while conformational fluctuations propagate.

Recognizing the lack of a standard protocol to rigorously account for proton-linked ligand binding to protein, we recently developed a protocol utilizing CpHMD to compute pH-dependent binding free energies [89]. In our computational method, the binding polynomial formalism devised by Wyman [90] is applied with the CpHMD framework to obtain a pH-dependent correction to a reference free energy of binding obtained at a given level of pH (ΔG°_ref,pH_). The proton-linked binding free energy then can be expressed according to Eq 8 provided in Methods, using the notation used by Tanford [91], where the total charges of the protein-ligand complex, protein, and ligand in Eq 8 are obtained from the CpHMD simulations. The integral in the second term in Eq 8 provides a thermodynamic relation that holds for pH-dependent ligand binding in cases where proton binding to different titratable sites may be cooperative (*i*.*e*., no assumptions are made about sites titrating independently). When applied to binding of small molecules to the cucurbit [7]uril host, this CpHMD-based free energy method accurately obtained the pH-dependent binding free energy profiles. Also, the method demonstrated that the traditional use of fixed protonation states for both free and bound states predicted based on pH 7 in free energy computations could give errors larger than 2 kcal/mol in the host-guest systems with a single titratable site [89]. Given the complexity of protein environment where multiple titratable groups exist, the corresponding error in free energy may be even larger in protein-ligand binding, highlighting the significance of accurate description of the binding-induced pK_a_ shifts in free energy computations.

**Fig 2:**
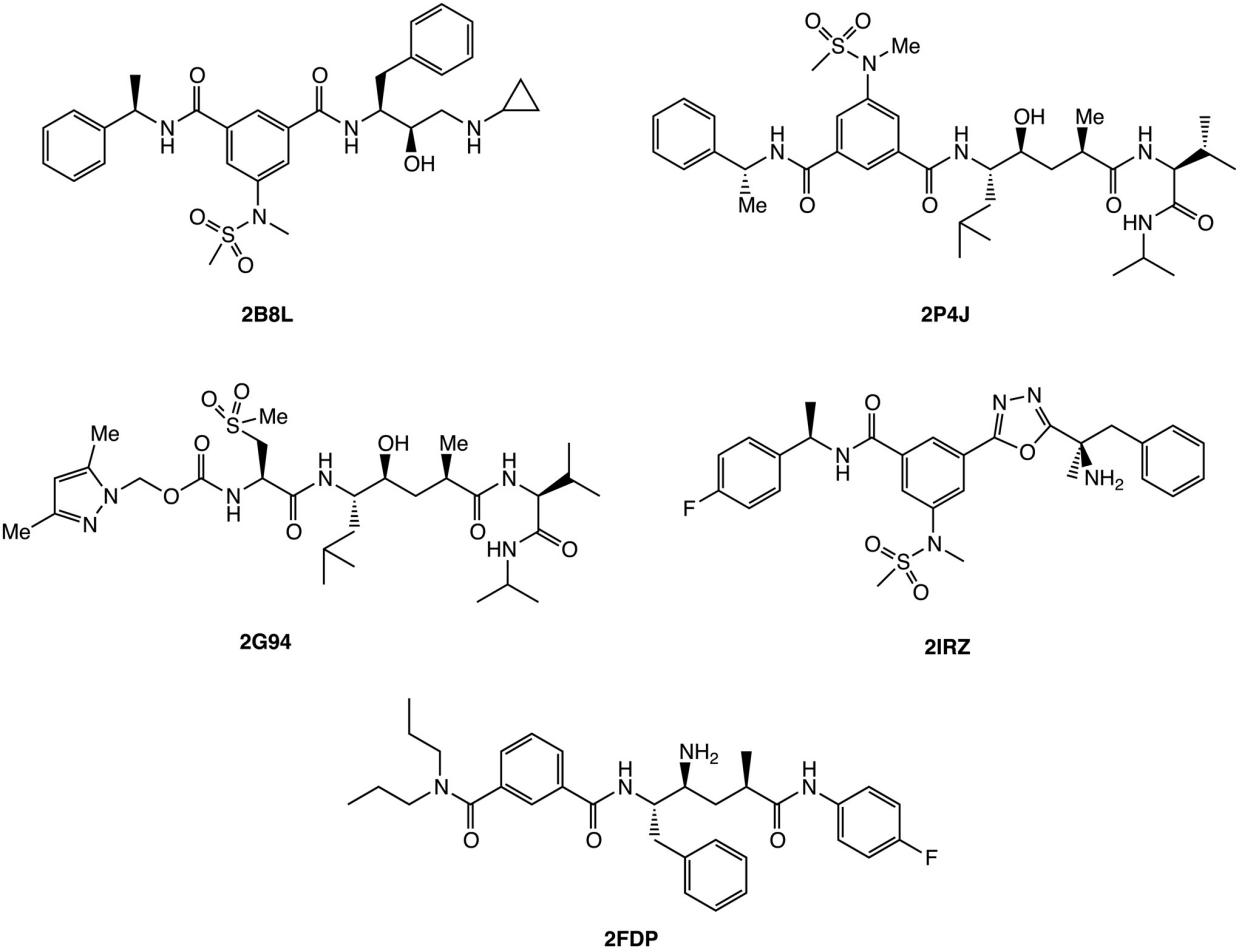
Chemical structures of BACE-1 inhibitors considered in this study. The PDB IDs are shown for complex structures of BACE-1 bound with respective inhibitors.

In this work, we have performed constant pH replica exchange molecular dynamics (pH-REMD) simulations to study the proton-linked conformational dynamics and binding free energies of inhibitors to BACE-1. The conformational changes of the flap region of BACE-1 in the absence and presence of inhibitors shown in Fig 2 are analyzed with respect to solution pH, which is found to act as a conformational switch. The microscopic pK_a_ values of ten titratable residues including the aspartyl dyad in BACE-1-inhibitor complexes are obtained from the pH-REMD simulations and compared with those computed for free enzyme. The results show significant binding-induced shifts in the pK_a_ values. We further apply our CpHMD-based computational protocol to these results, computing the pH-dependent binding free energy profiles of various inhibitors to BACE-1. The results demonstrate that incorrect assignment of protonation state to the titratable groups can result in errors of over 8 kcal/mol in free energy computations for the systems considered here, highlighting the significance of correctly addressing the binding-induced protonation changes. To the best of our knowledge, this work presents the first application of CpHMD simulations to quantify binding in protein-ligand systems and shows high utility for addressing pH effects in computer-aided drug discovery workflows.

## Results

### pH-Dependent Conformational Dynamics of Apo BACE-1

As conformational transitions of BACE-1 are suggested to play a role in catalysis [29–31], we first examine the dynamics intrinsic to apo BACE-1 before exploring the effect of inhibitor binding in the following section. Conventional molecular dynamics (cMD) simulations of duration 100 ns are carried out prior to constant pH molecular dynamics (pH-REMD) simulations in order to provide equilibration phase for apo and three inhibitor-bound systems prepared by homology modeling (see Methods). Using the protonation states assigned using the PROPKA program [75,92–94], the cMD simulations provide a benchmark for comparing pH-REMD simulations.

**Fig 3:**
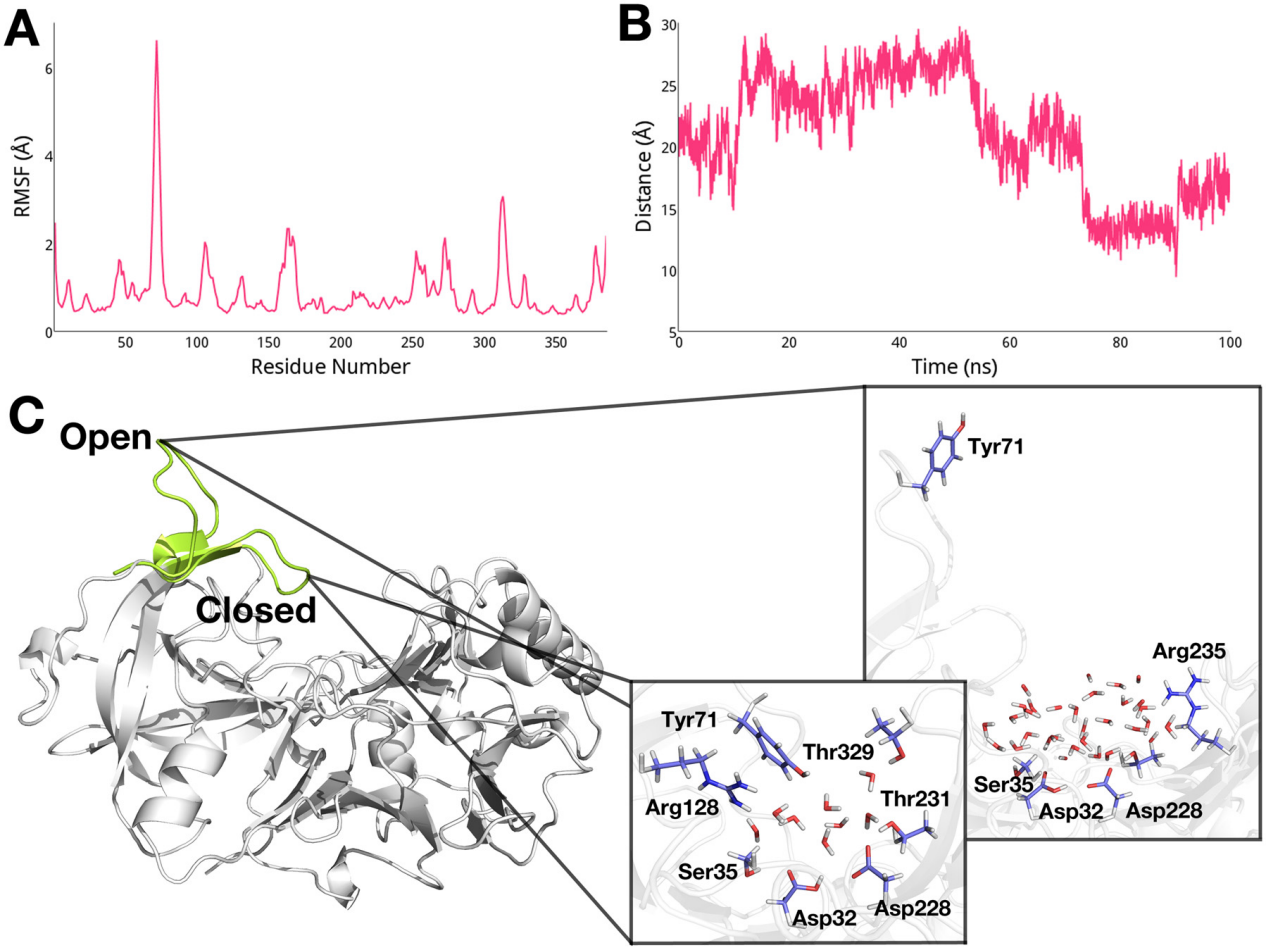
(A) RMSF of apo BACE-1 from the cMD simulations. (B) Change in distance between the dyad and Tyr71 in the flap region. (C) Open and closed conformations of the flap and interactions between the dyad and surrounding residues in each conformation.

In order to quantify the extent to which each residue fluctuates, we compute the root-mean-square fluctuation (RMSF) of each residue in apo BACE-1 from the cMD trajectory. As shown in Fig 3A, higher RMSF values are noted for the flap region (residues 67 to 77), consistent with the suggestion by others [29,31–34]. Taking a closer look at the flap region, we measure the distance between the center of mass of the aspartyl dyad and Tyr71, which is located at the tip of the flap region. From the change of the dyad-flap distance plotted in Fig 3B, it is evident that the flap region undergoes transitions between open and closed conformations, within the distance range of 10 Å (closed) to 30 Å (open). In the closed conformation of the flap, we observe a water-mediated hydrogen bond network that includes the dyad, Ser35, Tyr71, Arg128, Thr231, and Thr329 (Fig 3C), agreeing with the findings from previous studies [23,31,34]. On the other hand, when the flap is open, the dyad forms contacts mediated by water with Ser35, Thr231, and Arg235, while Tyr71 is entirely exposed to solvent (Fig 3C).

**Fig 4:**
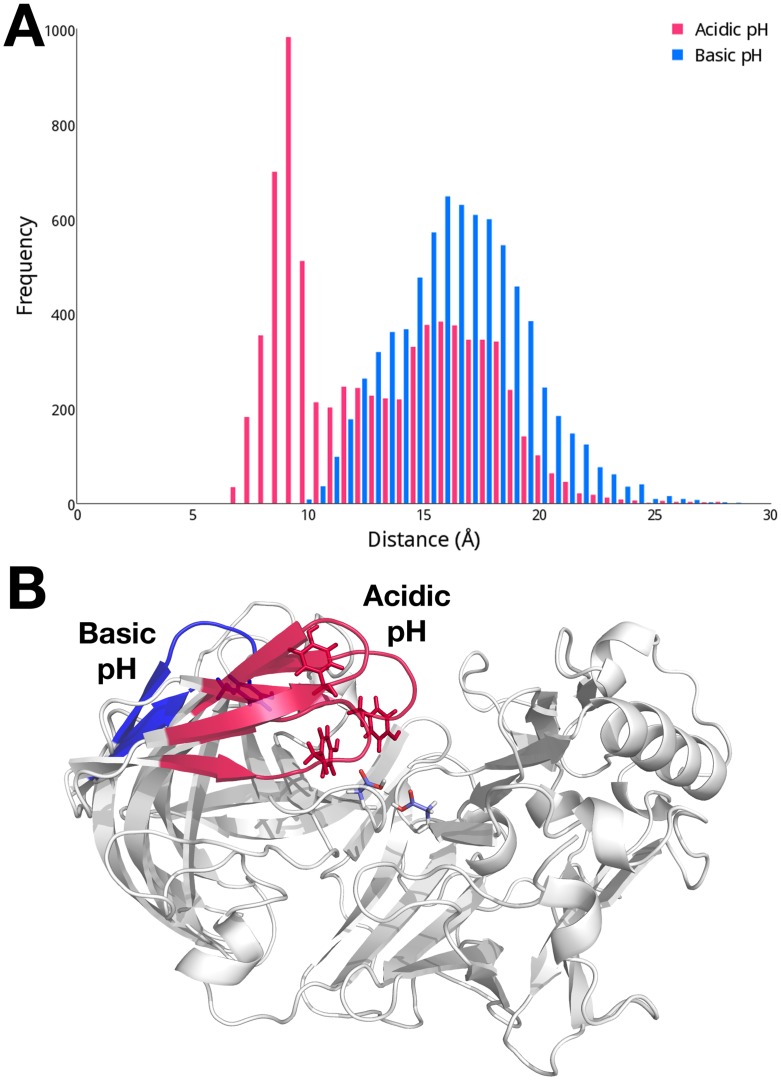
(A) Distributions of distances between the dyad and Tyr71 in apo BACE-1 at acidic (pH 1 to 3; red) and basic (pH 9 to 11; blue) pH. (B) Conformations of the flap region in the cluster representative structures of apo BACE-1 at acidic and basic pH. The distances between the dyad and flap of the representative structures are 9.17 Å, 11.0 Å, and 15.7 Å at acidic pH; and 17.6 Å at basic pH.

While the cMD simulation is performed with fixed protonation states, we choose to examine the effect of protonation equilibria on the conformational flexibility of apo BACE-1 as the enzymatic activity of BACE-1 is shown to be pH-dependent [24,25]. We focus this investigation on a comparison of the dynamics of the flap region at acidic (pH 1 to 3) and basic (pH 9 to 11) pH levels. The conformational space of the flap region sampled at these differing levels of pH is quantified by measuring the distances between the center of mass of the dyad and Tyr71 (Fig 4A). Since the pH-based trajectories reconstructed from the pH-REMD simulations are not time-dependent, distributions of the measured distances are presented here. The distance between the dyad and Tyr71 exhibits a bimodal distribution at acidic pH, with the flap sampling both open and closed states. When the flap is closed, an average distance between the dyad and flap is about 8 Å, while open conformations are also populated, having an average dyad-flap distance of ~ 17 Å. In order to visualize structural characteristics typical at varying solvent environments, we carry out a clustering analysis based on pairwise RMSDs of Cα atoms of the conformations sampled at acidic and basic pH. We find three dominant conformers that encompass 86% of total conformations sampled at acidic pH. The flap regions from representative structures of these three clusters are shown in red in Fig 4B, further illustrating the flap region sampling both open and closed conformations at acidic pH.

At basic pH, the flap exhibits noticeably different dynamics compared to acidic pH. The flap region remains over 10 Å away from the dyad and most frequently found in the open conformation with a distance of about 17 Å from the dyad (Fig 4A). From the clustering analysis, a single conformer is found to represent 82% of total conformations sampled at basic pH. In this typical conformer, the flap is in widely open state with a distance of 17.6 Å from the dyad and completely exposed to the solvent area, as shown in blue in Fig 4B.

### Conformational Dynamics of BACE-1 in Complex with Inhibitors

**Fig 5:**
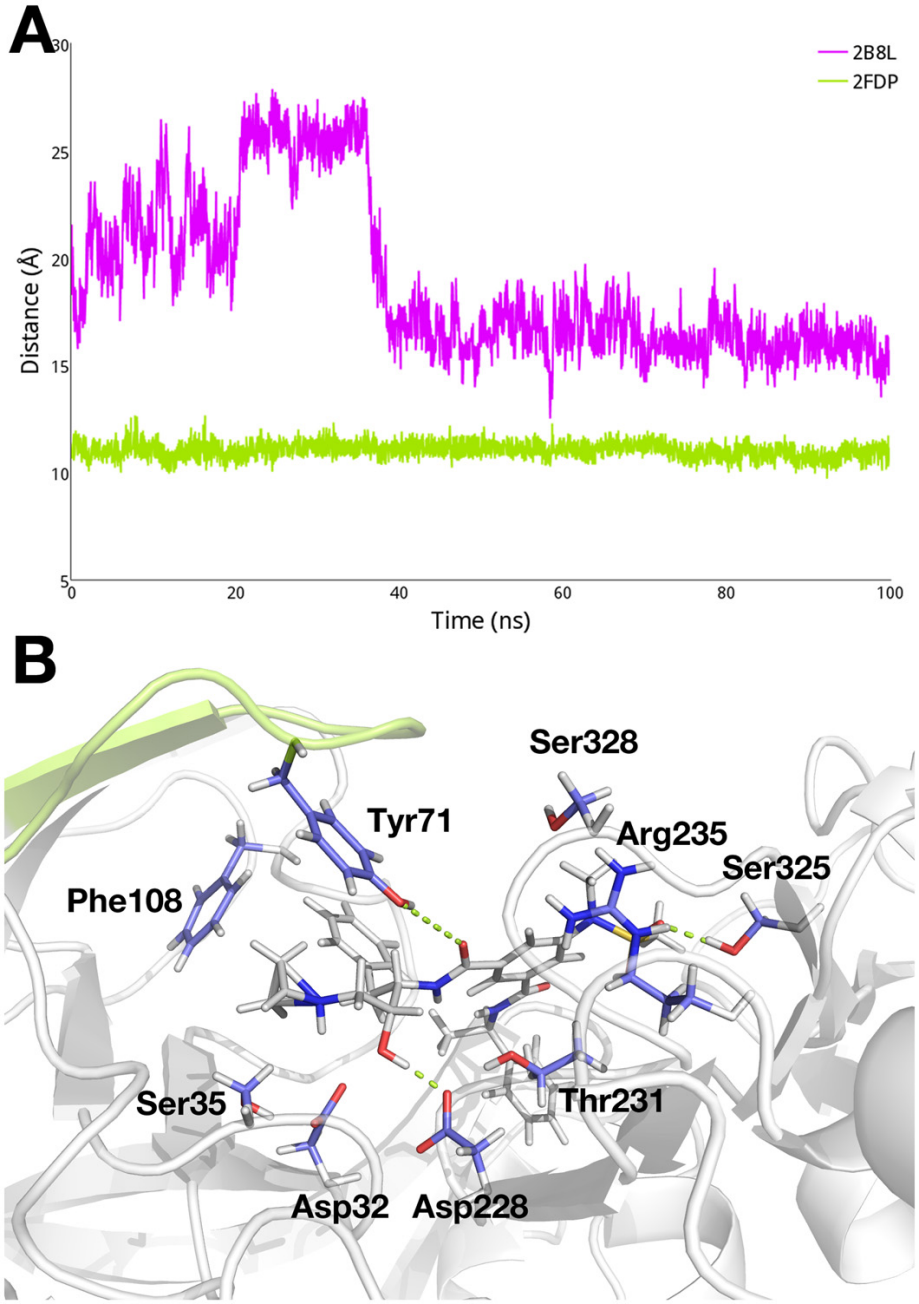
(A) Change in distance between the dyad and flap region in the 2B8L (purple) and 2FDP (green) systems in the respective cMD simulations. (B) Interactions between the bound inhibitor in the 2B8L complex and surrounding residues.

We continue to probe the changes in dynamics of BACE-1 that accompany inhibitor binding. While the shifts between open and closed conformations of the flap region are observed in the cMD simulations of apo BACE-1, the flap remains in a closed state in the inhibitor-bound cMD simulations. The distances between the center of mass of the dyad and Tyr71 observed in the cMD simulations of BACE-1 in complex with the inhibitors 2B8L and 2FDP, respectively, are shown in Fig 5A. In comparison to the distribution of distances observed in the cMD simulation of apo BACE-1 (Fig 3B), we observe significantly less flexibility in the flap region in the 2B8L and 2FDP systems. Fluctuation of the dyad-flap distance in the 2B8L system during the early stage of the simulation is likely due to structural instability arising from the homology modeling. However, after 40 ns, the flap region in the 2B8L complex achieves a stable state and remains closed at a distance of about 15 Å from the dyad. It is worth noting that the measured dyad-flap distances in the inhibitor-bound systems are inherently longer than that in apo enzyme; for instance, the open conformation of the flap in apo BACE-1 has the dyad-flap distance of ~ 15 Å whereas the closed conformation of the flap in the 2B8L system has the distance of 15 Å as well. This is because the flap is unable to penetrate into the active site as deep as in apo BACE-1, due to the presence of the bound inhibitors in the binding site. Similarly, the flap region in the 2FDP system maintains an average dyad-flap distance of ~ 11 Å. The bound inhibitors have hydrophobic interactions with Tyr71 and Phe108 while forming hydrogen bonds with the polar residues including the dyad, Tyr71, Thr231, and Ser325. These hydrogen bond networks effectively lock the flap in the closed state, as shown in Fig 5B for the 2B8L system. Similar trends in the dyad-flap distance are observed in other inhibitor-bound systems (S1 Fig).

**Fig 6:**
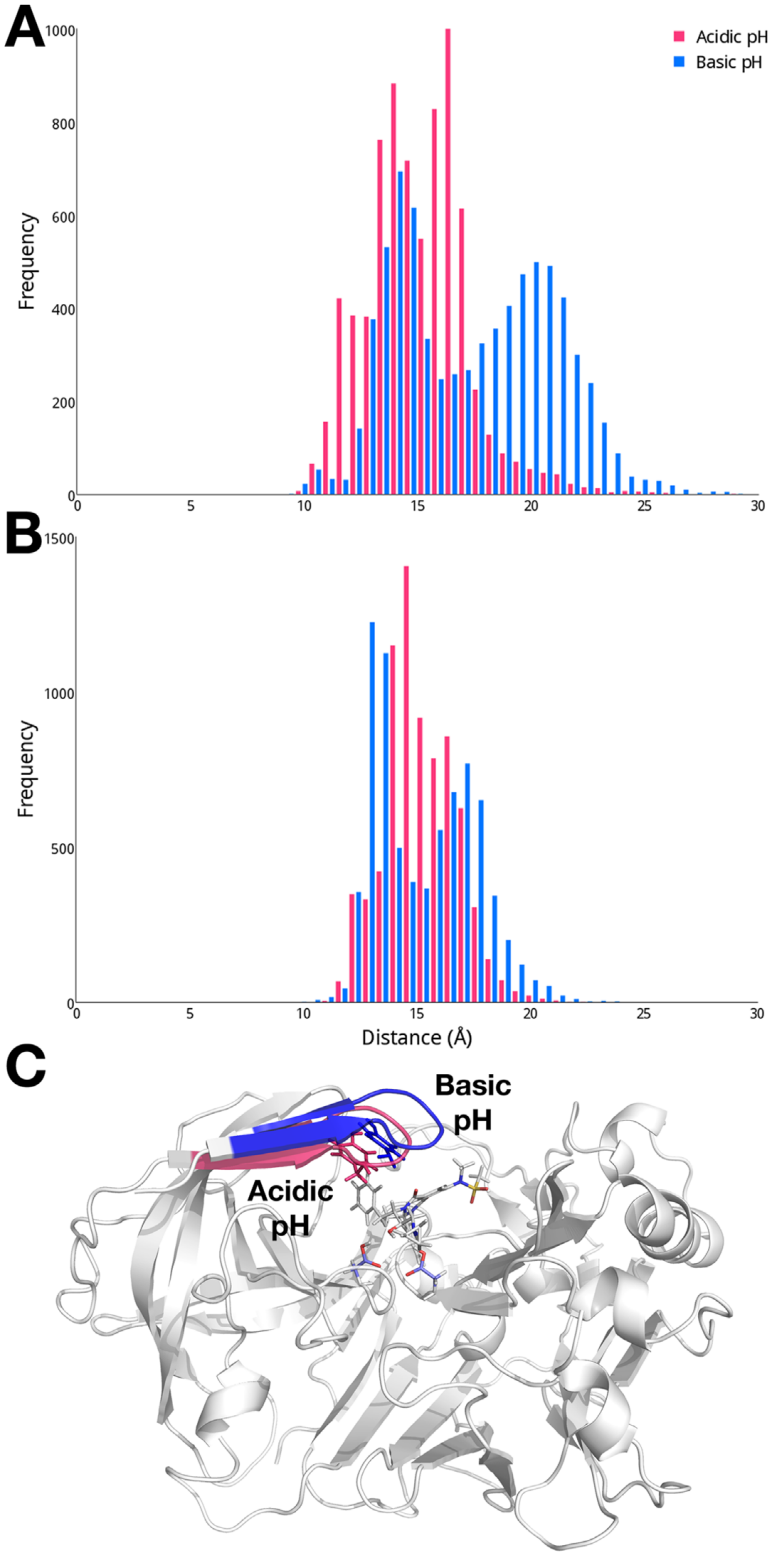
Distribution of distances between the dyad and flap in the 2B8L system at acidic (pH 1 to 3; red) and basic (pH 9 to 11; blue) pH. (A) 2B8L. (B) 2FDP. (C) Conformations of the flap in the cluster representative structures of the 2B8L system at acidic and basic pH.

As we observe the pH-controlled dynamics of the flap region in apo BACE-1, we also carry out similar analyses on the holo systems to examine the effect of pH on conformations of the flap in the presence of inhibitors. Unlike the apo system, a clear distinction in the dynamics of BACE-1 contingent on pH levels is not found in the inhibitor-bound systems. The distances between the center of mass of the dyad and Tyr71 indicate that a closed state of the flap is dominant in the 2B8L system at both pH, with average distances of ~ 15 Å, as well as slightly open conformation of the flap with the dyad-flap distance of ~ 19 Å at basic pH (Fig 6A). From the clustering analysis, the flap is in closed state in the typical structure representing 67 to 77% of the total conformations sampled at both pH conditions, as shown in Fig 6C, forming similar contacts with the bound inhibitor and dyad as observed in the cMD simulations (Fig 5B). Other cluster representative structures also exhibit similarly closed conformations the dyad-flap distances varying in the range of 15 to 19 Å. In the 2FDP system, the flap region exhibits essentially identical dynamics; the flap is in closed state at acidic pH and has both slightly open and closed conformation at basic pH. On average, the flap is found primarily in a closed conformation where the dyad-flap distance is 15.2 Å (Fig 6B). Consistent with these findings, the cluster representative structures of the 2FDP system at both acidic and basic pH have the flap in closed state, similar to the 2B8L complex (Fig 6C). Similar trends in the distributions of the dyad-flap distance in varying pH conditions are observed in other inhibitor-bound systems (S2 Fig).

### Computation of pK_a_ Values of Apo BACE-1

We further investigate the acidic properties of the aspartyl dyad and surrounding titratable sites in both apo and holo BACE-1. The pK_a_ values of the different titratable residues considered are obtained by fitting the Hill equation (Eq 3; Methods) to titration data obtained from the pH-REMD simulations conducted at different levels of pH (Table 1). We first analyze the computed pK_a_ values of the titratable groups in apo BACE-1 and compare the values obtained for inhibitor-bound systems in the following section.

**Table 1:** pK_a_ values obtained from pH-REMD simulations. The corresponding Hill coefficients are shown in parentheses.

	Apo	2B8L	2P4J	2G94	2IRZ	2FDP
Asp32	5.0 ± 0.2 (0.51)	9.7 ± 0.2 (1.72)	8.7 ± 0.6 (0.34)	9.54 ± 0.05 (2.19)	8.4 ± 0.7 (0.40)	8.9 ± 0.7 (0.33)
Asp106	3.99 ± 0.01 (1.05)	3.94 ± 0.01 (0.89)	4.00 ± 0.01 (0.97)	3.91 ± 0.01 (0.97)	3.85 ± 0.01 (0.96)	3.85 ± 0.01 (0.99)
Asp138	6.26 ± 0.01 (1.16)	6.10 ± 0.02 (0.93)	5.59 ± 0.04 (0.86)	5.72 ± 0.01 (0.92)	6.16 ± 0.01 (1.04)	6.40 ± 0.01 (1.11)
Asp223	5.20 ± 0.03 (0.91)	5.99 ± 0.03 (1.07)	5.03 ± 0.02 (0.92)	5.34 ± 0.01 (0.95)	4.53 ± 0.03 (1.02)	5.46 ± 0.02 (1.06)
Asp228	5.9 ± 0.5 (0.41)	8.4 ± 0.1 (1.26)	8.3 ± 0.1 (1.75)	8.1 ± 0.2 (0.90)	8.3 ± 0.1 (0.79)	9.7 ± 0.6 (0.30)
Glu116	7.23 ± 0.02 (1.02)	7.9 ± 0.1 (0.77)	7.51 ± 0.04 (0.86)	7.08 ± 0.01 (1.26)	7.42 ± 0.04 (0.77)	8.27 ± 0.09 (1.34)
Glu265	4.31 ± 0.01 (0.96)	4.39 ± 0.01 (0.95)	4.28 ± 0.01 (0.95)	4.32 ± 0.01 (0.97)	4.32 ± 0.01 (0.98)	4.33 ± 0.01 (0.98)
Glu339	6.6 ± 0.1 (0.73)	7.01 ± 0.07 (0.88)	7.46 ± 0.02 (1.01)	6.16 ± 0.03 (1.03)	7.35 ± 0.02 (1.66)	6.88 ± 0.02 (1.00)
His45	5.6 ± 0.1 (1.15)	4.3 ± 0.1 (0.91)	6.33 ± 0.02 (1.06)	6.26 ± 0.02 (1.16)	5.99 ± 0.08 (0.87)	5.95 ± 0.02 (1.17)
Tyr71	9.68 ± 0.01 (1.02)	9.4 ± 0.3 (1.05)	9.62 ± 0.02 (1.13)	9.71 ± 0.03 (1.99)	9.4 ± 0.03 (0.86)	9.92 ± 0.09 (0.85)

**Fig 7:**
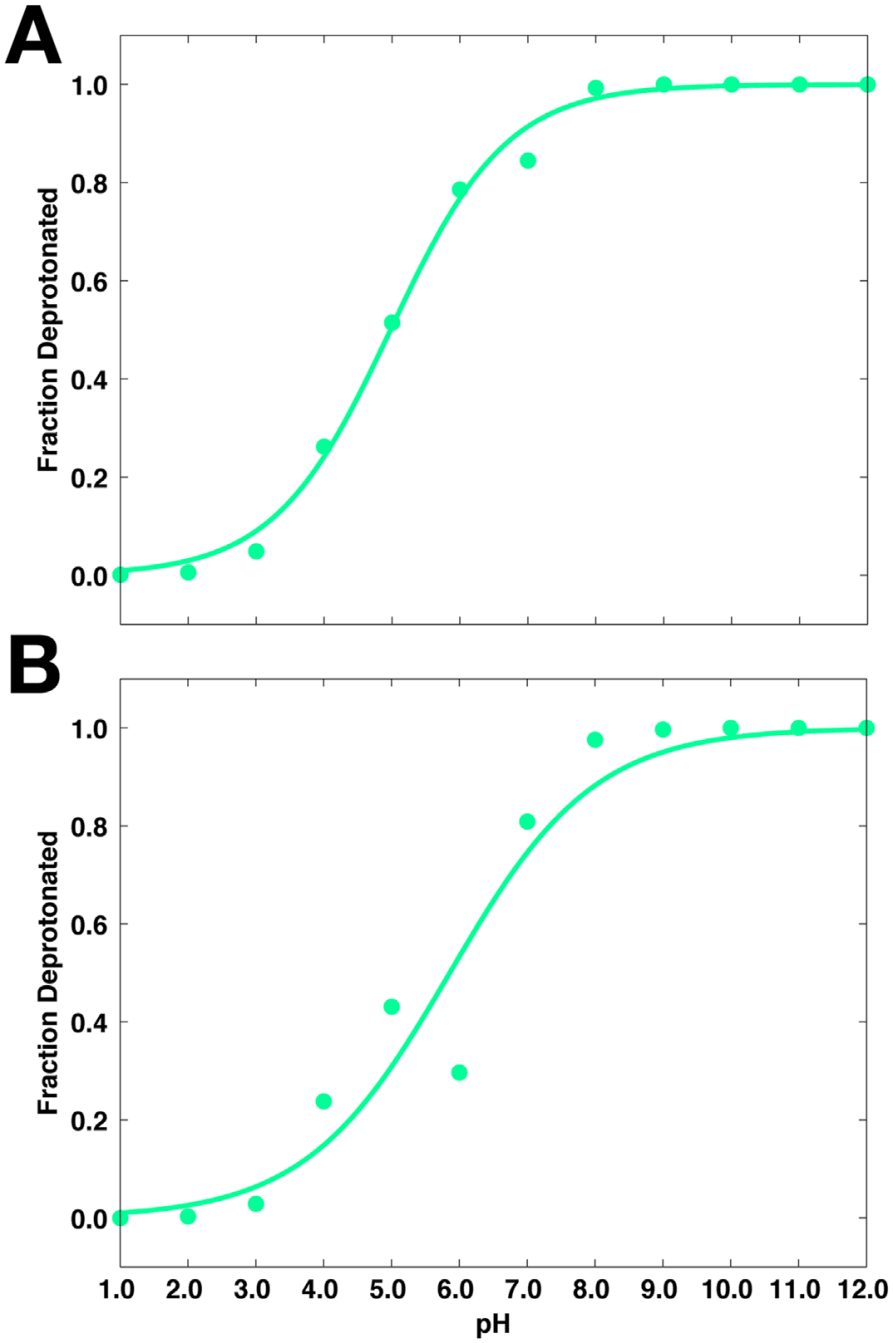
Titration curves from the pH-REMD simulations of apo BACE-1. (A) Asp32. (B) Asp228.

The predicted pK_a_ values of the aspartyl dyad, Asp32 and Asp228, are 5.0 ± 0.2 and 5.9 ± 0.5, respectively. Titration curves for the dyad are shown in Fig 7, plotted as fraction of deprotonated species of each residue as a function of pH. From the titration curves, both aspartates are completely protonated at acidic pH levels (pH < 3), while fully deprotonated at basic pH (pH > 8). Between pH 4 and 8, Asp32 and Asp228 exist in an ensemble of protonated and deprotonated forms. To illustrate this, Asp32 and Asp228 are approximately 20% and 40% deprotonated, respectively, at pH 4.5, the pH at which BACE-1 is most active. These observed shifts from the typical pK_a_ of Asp residues (4.0) [95,96] may aid in the proton transfer step required in BACE-1 catalysis. The Hill coefficients of the dyad deviate from one, suggesting that titration of Asp32 and Asp228 is coupled. The coupled titration observed for these residues also contributes to greater noise in their respective titration curves, leading to larger errors in in their pK_a_ (Table 1).

The pK_a_ values of the remaining titratable residues are also reported in Table 1. All ionizable residues besides the dyad appear to titrate independently of each other, as suggested by their Hill coefficients that are approximately one. Furthermore, the statistical errors from fitting procedure to obtain the pK_a_’s of these residues are minimal. Similar to those of the dyad, the computed pK_a_ values of Asp138, Asp223, Glu116, and Glu339 are shifted higher than the canonical pK_a_ values for Asp (4.0) and Glu (4.4) [95,96], whereas Asp106 exhibits titration behavior in line with model Asp. As these residues are distant from the active site, these deviations in the computed pK_a_ values arise mainly from the microenvironments surrounding them. For instance, Glu116 and Glu339 are buried in the protein interior (Fig 1), where the microscopic dielectric constants can be different from that of the bulk solvent [43,44,97,98]. In order to compensate for the desolvation energy, the neutral, protonated forms of the glutamates are favored in the protein interior at the solution pH where they would normally be charged if they were not buried. Titration curves for the remaining titratable residues are provided in S3 Fig.

### Binding-Induced pK_a_ Shifts of Titratable Residues in BACE-1

**Fig 8:**
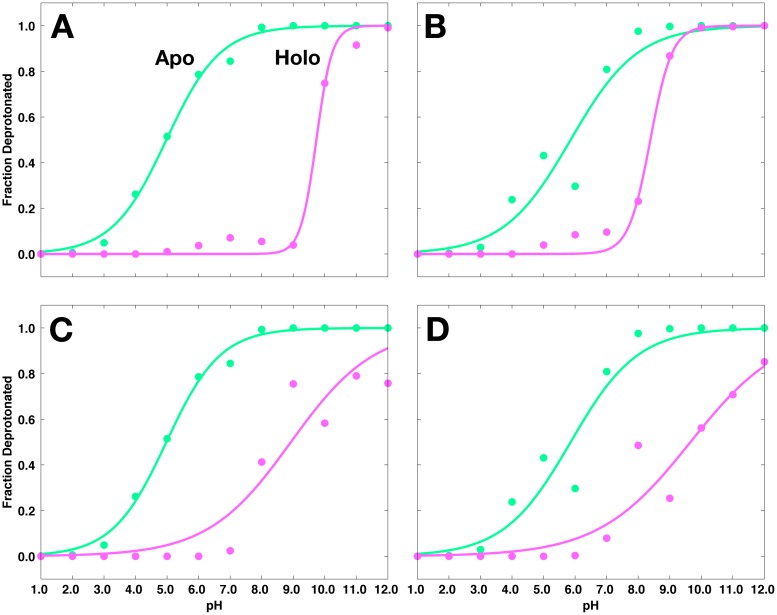
Titration curves for the aspartyl dyad in the 2B8L and 2FDP systems, shown in purple. (A) Asp32, (B) Asp228 in the 2B8L system; (C) Asp32, (D) Asp228 in the 2FDP system. Respective titration curves of the dyad in apo BACE-1 are shown in green.

Having observed the pK_a_ values of various titratable groups in apo BACE-1, we shift our attention to the inhibitor-bound systems. First considering the dyad, the pK_a_ values of Asp32 and Asp228 are shifted toward more basic values of pH when various inhibitors are present, with computed pK_a_ values greater than 8.1. Representative titration curves for the dyad in 2B8L and 2FDP systems (purple curves) are compared with those in apo BACE-1 (green curves) in Fig 8. Similar curves corresponding to other inhibitor-bound systems are shown in S4–S6 Figs.

In the 2B8L system, Asp32 and Asp228 have pK_a_ values of 9.7 ± 0.2 and 8.4 ± 0.1, respectively (shifted + 4.7 and + 2.5 pK units relative to their values in apo BACE-1). From the titration curves in Fig 8A and 8B, it is apparent that both Asp32 are Asp228 are significantly protonated under pH 8–9 in the 2B8L system. Above this pH, the dyad exists in an ensemble of different protonation states between pH 8 and 10. Similar binding-induced pK_a_ shifts are observed for the dyad in 2FDP system. In this case, the computed pK_a_’s of Asp32 and Asp228 are 8.9 ± 0.7 and 9.7 ± 0.6, respectively. As shown in Fig 8C and 8D, in the 2FDP complex, both aspartates are predominately protonated at the pH levels below 7, and exist as an ensemble of different protonated forms at basic pH. In both cases, as in the apo enzyme, we observe the coupled titration behavior of the dyad and relatively large errors during the fitting of titration data to the Hill equation.

The remaining titratable groups examined, all of which are distant from the binding site, do not undergo pK_a_ shifts larger than 1.3 pK units upon inhibitor binding (S4–S8 Figs). The narrow pK_a_ shifts of these titratable groups upon complex formation suggest that binding of inhibitors to BACE-1 is thermodynamically linked to a proton transfer that is primarily localized at the catalytic dyad.

### pH Dependence of the Binding Free Energy of Inhibitors

The large shifts in pK_a_’s of the aspartyl dyad upon binding of inhibitors to BACE-1 indicate that proton binding is linked to complex formation in BACE-1 systems. Utilizing our recently developed computational protocol [89], we present the pH-dependent changes in free energies of binding of inhibitors to BACE-1. Application of the binding polynomial formalism to the results obtained from the pH-REMD simulations provides pH-dependent corrections to the reference binding free energies obtained for a given pH, ΔG°_ref,pH_ (Eq 8). The reference free energies of binding of inhibitors for BACE-1-inhibitor systems are obtained from experimental association constants measured at pH 4.5 [99–102].

**Fig 9:**
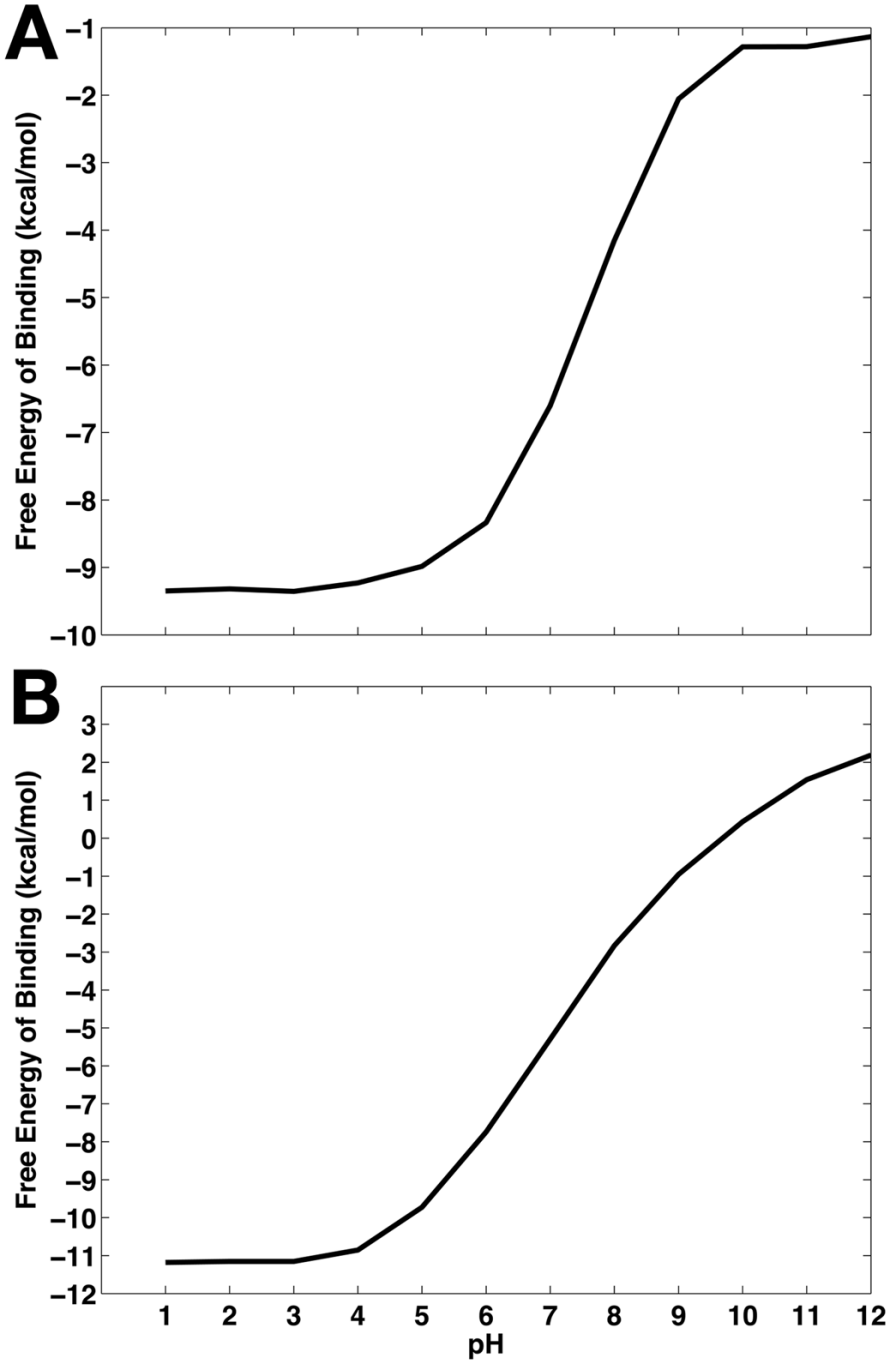
Binding free energy profiles of inhibitors as functions of pH. (A) 2B8L. (B) 2FDP.

Binding free energy profiles as functions of pH of the 2B8L and 2FDP complexes are shown in Fig 9. Significant changes in binding free energies from the reference free energies at pH 4.5 are observed as solution pH increases. Considering the 2FDP complex, we see that binding is most favorable at acidic pH, where the maximum affinity is -11.2 kcal/mol. As pH increases, binding becomes less favorable. This is most pronounced in the pH range of 5 to 10, where the aspartyl dyad that interacts directly with the bound inhibitor begins to populate deprotonated states, leading to an ensemble of protonated and deprotonated species of the dyad at the pH levels between 5 and 10 (Fig 8B). As the deprotonated forms of the dyad develop, hydrogen bonds made between the diprotonated dyad and bound inhibitors at low pH are lost, and consequently, the binding affinity becomes weaker in this pH range and is least favorable with an affinity of 2.2 kcal/mol at pH 12. Similar binding free energy profiles as functions of pH for other inhibitor-bound systems can be found in S9 Fig.

In Table 2, the binding free energies are compared in the pH ranges that are most relevant to biological conditions. The binding free energy for the 2FDP system changes by 5.1 kcal/mol between pH 4.5 and 7. Comparison to the binding free energy at pH 10 leads to more dramatic changes; for instance, the binding affinity differs by 10.8 kcal/mol between pH 4.5 and pH 10 for the 2FDP complex. As mentioned above, such large changes are due to the shifts in protonation state of the dyad at these pH levels from the diprotonated state observed at pH 4.5, and further highlight the significance of correctly accounting for the protonation states of the titratable groups which accompany a net proton transfer upon inhibitor binding.

**Table 2:** Binding free energies of the inhibitors upon complex formation with BACE-1. All energies are reported in kcal/mol.

	ΔG°_ref, pH_ (pH 4.5)	ΔG° (pH 7)	ΔΔG°_pH7-ref_	ΔG° (pH 10)	ΔΔG°_pH10-ref_
2B8L	-9.1 [99]	-6.6	2.5	-1.3	7.8
2P4J	-12.3 [99]	-8.7	3.6	-4.0	8.3
2G94	-13.1 [100]	-9.4	3.7	-4.9	8.2
2IRZ	-10.1 [101]	-6.1	4.0	-2.6	7.5
2FDP	-10.4 [102]	-5.3	5.1	0.4	10.8

## Discussion

The dependence of BACE-1 enzymatic activity on solution pH and the need for conformational change to accompany the catalysis are both well established [24,25,30,31,34]. As a promising therapeutic target for the treatment of Alzheimer’s disease, understanding the detailed mechanism underlying the pH dependence of BACE-1 dynamics and enzymatic activity is imperative for structure-based drug design. In this work, we have performed constant pH replica exchange molecular dynamics (pH-REMD) simulations to examine the proton-linked conformational dynamics and inhibitor binding properties of BACE-1.

Significant flexibility of the flap region (residues 67 to 77), resulting in transitions between open and closed conformations, is noted for apo BACE-1 during conventional molecular dynamics (cMD) simulations (Fig 3). As we further probe the effect of pH on conformational flexibility of apo BACE-1, distinctive conformations that are characteristic of different pH environments are captured from the pH-REMD simulations. At acidic pH, both open and closed conformations of apo BACE-1 are significantly populated, whereas a single conformer with the flap closed predominates in basic conditions (Fig 4).

While we observe the flexible nature of apo BACE-1, the presence of inhibitors at the active site of BACE-1 greatly reduces conformational mobility of the enzyme (Fig 5). The bound inhibitors form various hydrophobic and polar interactions with the surrounding residues, holding the flap in a tightly closed state. Similarly closed conformations of the flap are observed regardless of the varying pH conditions, indicating that the structural flexibility of BACE-1 is largely limited by the presence of bound inhibitors.

We determine the microscopic protonation states of the dyad residues and surrounding titratable residues to further probe the mechanism underlying the pH dependence of the catalytic activity of BACE-1. First, the pK_a_ values of ten titratable residues in various BACE-1 systems are obtained from the pH-REMD simulations (Table 1). The computed pK_a_ values of Asp32 and Asp228 in apo BACE-1 are 5.0 ± 0.2 and 5.9 ± 0.5, respectively, shifted from the typical pK_a_ of Asp (Fig 7). At acidic pH, protonated states are dominant for both aspartates of the dyad. This allows the dyad to form hydrogen bonds with the flap residues, *i*.*e*., Tyr71 and Thr72 and allows for closed conformations to be sampled at low pH. On the other hand, open conformations of the flap are also populated at acidic pH; these open conformations likely aid in substrate binding and product release in the course of catalysis [29–31]. When we solvate the open and closed conformers from the pH-REMD simulations, which are performed in implicit solvent, similar water occupancies to those in the cMD simulations are observed (Fig 3C). When the flap is open, the active site becomes largely accessible to water, which is needed to act as nucleophile for the hydrolytic catalysis by BACE-1. Also, water molecules entering the active site help to compensate for the breaking of hydrogen bonds between the dyad and flap by forming alternative hydrogen bonds and mediating the hydrogen bond networks with surrounding charged residues such as Ser35 and Arg235. Therefore, conformational transitions between open and closed states of the flap at acidic pH allow for channeling of solvent, substrates, and hydrolytic products to and from the active site in catalysis. With the pK_a_ values near the pH of optimal enzymatic activity, *i*.*e*., pH 4.5, the dyad is also able to easily gain and release proton(s) during the catalytic cycle. Hence the pK_a_ values of the dyad of BACE-1 shifted from the typical value may be an evolutionary result to achieve the maximal activity at pH 4.5.

As pH increases, deprotonated species of both the dyad and Tyr71 start to emerge. Consequently, the hydrogen bond networks observed in acidic conditions no longer persist and are instead replaced by water molecules, leading to the primarily open conformation of the flap at basic pH. While this open state of the flap is stabilized energetically through water-dyad interaction, it is likely that the persistence of the open conformation at basic pH disables the enzyme’s ability to corral the substrate into the binding site for catalysis. Therefore, observation of the invariably open state of the flap at basic pH is consistent with the suggested role of flexibility of flap in the catalysis at acidic pH [29–31]. Our results thus indicate that the conformational dynamics intrinsic to the enzymatic catalysis of BACE-1 are modulated by solution pH, further suggesting the enzyme’s structural adaptation during the evolution for its maximal activity.

The Hill coefficients for fitting the titration data to the Hill equation suggest the titration for the aspartyl dyad is cooperative in both free and complexed BACE-1, while independent, uncoupled titrations are observed for other residues considered. Such coupled titration behavior makes computing the microscopic pK_a_ values difficult. Hence, the statistical errors associated with computing the pK_a_’s of the dyad are higher than those of other titratable residues.

At pH 4.5, the aspartyl dyad exists in an ensemble of protonated and deprotonated species in apo BACE-1 (Fig 7). Upon binding of inhibitors, however, significant shifts in the pK_a_ values are observed for the dyad, with both Asp32 and Asp228 having pK_a_ values between 8 and 10 (Table 1). Inhibitor binding effectively alters the protonation state of the dyad at pH 4.5 to its diprotonated form in all cases studied here. The protonated forms are preferred for both aspartates in the presence of inhibitor. This diprotonated state likely compensates for the unfavorable energetics associated with desolvation upon inhibitor binding, allowing for hydrogen bonds between the dyad and the bound inhibitor. The minimal pK_a_ shifts observed for the remaining titratable residues of BACE-1 upon complex formation imply a thermodynamic linkage between inhibitor binding and proton transfer primarily localized at the dyad.

Among several computational efforts to determine protonation state of the aspartyl dyad in BACE-1, a recent work by Domínguez *et al*. also examined the pK_a_’s of buried titratable groups in the 2B8L and 2IRZ systems [40], whose computed values are compared with ours in Table 3. At first glance, the predicted pK_a_ values differ the most between the two works for Asp138 and Asp228. In Domínguez *et al*., the pK_a_ of Asp228 does not deviate much from pK_a,ref_ of Asp (4.0) in both systems compared, while Asp32 was predicted to undergo more significant pK_a_ shift relative to its dyad partner. On the other hand, pK_a_’s of both aspartates in the dyad shifted to more basic values of pH in our calculations.

**Table 3:** Comparison of computed pK_a_ values of the titrable groups in 2B8L and 2IRZ.

	2B8L		2IRZ	
	This work	Domínguez *et al*. [40]	This work	Domínguez *et al*. [40]
Asp32	9.7 ± 0.2	8.35	8.4 ± 0.7	6.88
Asp138	6.10 ± 0.02	3.23	6.16 ± 0.01	3.38
Asp228	8.4 ± 0.1	4.47	8.3 ± 0.1	4.01
Glu116	7.9 ± 0.1	5.95	7.42 ± 0.04	6.09
Glu339	7.01 ± 0.07	6.10	7.35 ± 0.02	6.26

When comparing the results in Table 3, several distinctions in the methods used in two studies for pK_a_ prediction should be noted. First, the GB-OBC implicit solvent model [103] was used in Domínguez *et al*. [104] while the GB-Neck 2 model, in which improved results have been obtained with the added parameters to the GB-OBC [105], was employed in the pH-REMD method used here. Also, the internal dielectric constant of 10 [104] was used in their work while the GB-Neck 2 implementation in Amber 14 employs 1 [105]. In addition to the difference in the force fields utilized, perhaps more importantly, the conformational changes upon inhibitor binding were not rigorously accounted for in their study. Although the detailed comparison of the algorithms used for pK_a_ calculation is beyond the scope of this work, addressing the conformational aspect is particularly important for studying BACE-1 due to its flexible dynamic nature. This is especially crucial when computing the pK_a_ values of the titratable groups in the absence of bound inhibitors, as the conformational fluctuations in the flap also imply the change in solvent accessibility. Hence, the dielectric response of the aspartyl dyad can be different in the presence and absence of bound inhibitors, which in turn can affect the computed pK_a_ values. Consequently, it is evident that conformational transitions accompanying binding of inhibitors should be accounted for in calculation of pK_a_ values of BACE-1. As all previous attempts to compute the pK_a_’s of the titratable groups in BACE-1 have been largely limited to the use of static X-ray crystallographic structures, our results obtained from concurrent sampling of protonation and conformational spaces by pH-REMD provide a new insight into the microscopic pK_a_ values of BACE-1.

Application of our recently developed constant pH molecular dynamics (CpHMD)-based computational protocol [89], which applies the binding polynomial formalism to address the pH dependence of binding free energies, enables us to obtain proton-linked binding free energy profiles of various inhibitors. As shown in Fig 9 and S9 Fig, all inhibitors bind most strongly at acidic pH. The changes in binding free energies are most pronounced in the pH range of 4 to 10, which essentially encompasses most biological reactions. The deviations in binding free energies within this pH range from the reference binding free energies at pH 4.5 arise from the shift in populations of major protonated species of the titratable residues, primarily those of the dyad. Between pH 5 and 10, the dyad starts to populate the deprotonated species (Fig 8), and as the deprotonated forms of the dyad develop, hydrogen bonds made between the diprotonated state of the dyad and bound inhibitors at low pH break. Subsequently, the binding free energies of the inhibitors become very unfavorable as pH increases. Such observations are impossible with cMD simulations where the protonation states are fixed and fractional protonation is not allowed. This highlights the benefit of using CpHMD method in order to address cases in which changes in protonation states are critical.

Furthermore, our results emphasize the importance of correctly addressing the binding-induced changes in protonation states in protein-ligand systems where binding accompanies a net proton transfer. In conventional molecular modeling or free energy computations, the protonation states of the titratable groups, which are set ahead of time, are fixed and assumed to be identical for both free and bound states. Consistent with this convention, consider a hypothetical scenario in which both Asp32 and Asp228 are assumed to be completely protonated in both apo and holo states. In the case of 2B8L system, such protonation state of the dyad will result in binding free energy of the inhibitor of -9.3 kcal/mol (Fig 9A). On the other hand, when both aspartates are considered fully deprotonated, the binding free energy of the inhibitor is -1.3 kcal/mol. In these two extreme scenarios where the identical, discrete protonation state of the dyad is assumed for both free and bound states, the binding free energies deviate from the true free energy in which the protonation states are considered separately for apo and holo states and fractional protonation is allowed. The errors are as large as 8 kcal/mol for the 2B8L system and similar deviations are noted for other inhibitor-bound systems considered here, ranging between 8 and 12.6 kcal/mol. Such errors are nontrivial and the magnitude is in great excess of typical errors from free energy computations [106,107].

In addition, we note the lack of binding free energies of inhibitors to BACE-1 that are experimentally measured at pH levels other than pH 4.5. For BACE-1, the inhibition assays are traditionally carried out at pH 4.5 where the catalytic activity of BACE-1 is maximal. However, from a free energy computational standpoint, it would be greatly beneficial if binding free energies were measured at other pH levels to incorporate the effect of pH into free energy computations. Availability of experimental reference binding energies at various pH will be of great importance to pushing the free energy computation field forward.

The results presented here demonstrate the dynamics of BACE-1 controlled by solvent pH. The flexible motions of the flap region at low pH, assisted by the diprotonated state of the aspartyl dyad, enable the enzyme’s optimal catalytic mechanism at acidic environment, implying a linkage between the protonation equilibria, conformational dynamics, and catalytic activity of BACE-1. In addition, we show the thermodynamic relation between binding of inhibitors and protons at the active site of BACE-1. Our results highlight the importance of accurately accounting for the protonation states of the titratable groups in protein-ligand systems where ligand binding is pH-dependent. Furthermore, we show that the CpHMD method can be used as an all-purpose tool to assess the pH-dependent dynamics and to quantify the binding free energies for protein-ligand systems where the protonation equilibria play an important role. To the best of our knowledge, this work presents the first application of our CpHMD-based free energy method to protein-ligand systems. In using the method, absolute binding free energies obtained by computational free energy calculations such as thermodynamic integration can be used in cases where experimental association constants are not available. Our results highlight high utility of CpHMD method to address the effect of pH on conformational dynamics and inhibitor binding in computer-aided drug discovery workflows.

## Methods

### Constant pH Replica Exchange Molecular Dynamics

Baptista *et al*. developed the constant pH molecular dynamics (CpHMD) method to enable concurrent sampling of discrete protonation states and conformational space according to the semi-grand canonical ensemble [78]. In this work, we apply the flavor of CpHMD coupled with replica exchange (pH-REMD) [108] implemented in the AMBER 14 suite of programs [95] with generalized Born (GB) electrostatics. In the CpHMD simulations, a conventional molecular dynamics (MD) simulation is periodically interrupted by a Monte Carlo (MC) step, in which a change in the protonation state of a random titratable residue is considered [81]. Acceptance of the new protonation state is contingent on the computed transition free energy, ΔG_trans_:

$$\Delta {\text{G}}_{\text{trans}}={\text{k}}_{\text{B}}\text{T}(\text{pH}-{\text{pK}}_{\text{a},\text{ref}})\text{ln}10+\Delta {\text{G}}_{\text{elec}}-\Delta {\text{G}}_{\text{elec},\text{ref}},$$ (1)

where pH enters as an external thermodynamic parameter and k_B_T is the Boltzmann constant multiplied by the temperature of the system. For a given value of pH, the difference in electrostatic free energy that accompanies the change in protonation being considered, ΔG_elec_, is computed with respect to the difference in electrostatic free energy that accompanies analogous change in protonation for a model compound, ΔG_elec,ref_, which has a known pK_a_ value (pK_a,ref_). As all titratable residues in this study are protein residues, the model compounds referenced in Eq 1 are individual amino acids in GB solvent. The respective pK_a,ref_ values for CpHMD in AMBER 14 are 4.0 for Asp, 4.4 for Glu, 6.5 and 7.1 for His, 9.6 for Tyr, and 10.4 for Lys [95]. Computing ΔG_trans_ with respect to these model compounds enables cancellation of non-classical terms. The Metropolis criterion is then applied to ΔG_trans_ to determine whether to accept the proposed change in protonation, and the MD simulation is resumed. Repeated application of these steps builds an ensemble of protonation states along the MD trajectory.

In pH-REMD, the exchange between adjacent replicas is achieved in the pH dimension at a fixed conformation, whose acceptance ratio is dependent on the MC exchange probability for replicas i and j:

$${\text{P}}_{\text{i}\to \text{j}}=\text{min}\left\{1,\text{exp}\left[\text{ln}10({\text{N}}_{\text{i}}-{\text{N}}_{\text{j}})({\text{pH}}_{\text{i}}-{\text{pH}}_{\text{j}})\right]\right\},$$ (2)

where N_i_ is the number of titratable protons in replica i and pH_i_ is the pH of replica i prior to the exchange attempt [108,109]. By enhancing the sampling through an application of replica exchange scheme, the method has been shown to achieve faster convergence and better sampling in both conformational and protonation spaces compared to original CpHMD [83,108].

From the pH-REMD simulations, the pK_a_ of a given residue is computed as the midpoint of titration by fitting titration data to the Hill equation:

$$\text{s }=\;\frac{1}{1+10^{{\text{n(pK}}_{\text{a}}-\text{pH)}}},$$ (3)

where s is the fraction of deprotonated species for a given residue and n is the Hill coefficient. The fraction of deprotonated species (s) for a titratable group is obtained at each value of pH from the pH-REMD simulations. In using Eq 8 shown below, the fractions of protonated species (1-s) of the protein-inhibitor complex and protein can be translated into Z_PL_ and Z_P_, respectively.

### Computing the pH Dependence of Protein-Ligand Binding Free Energies

The binding polynomial formalism devised by Wyman [90] was used by Tanford to study protein denaturation [91] and by several groups to examine the pH dependence of protein-protein binding [68,110]. Following their theoretical foundations, we recently applied it to binding of a general receptor to a ligand with a single titratable site, and the detailed derivation of the formalism can be found therein [89]. Here we briefly outline the theoretical basis of the method and show its usage for protein-ligand binding with multiple titratable sites.

**Fig 10:**
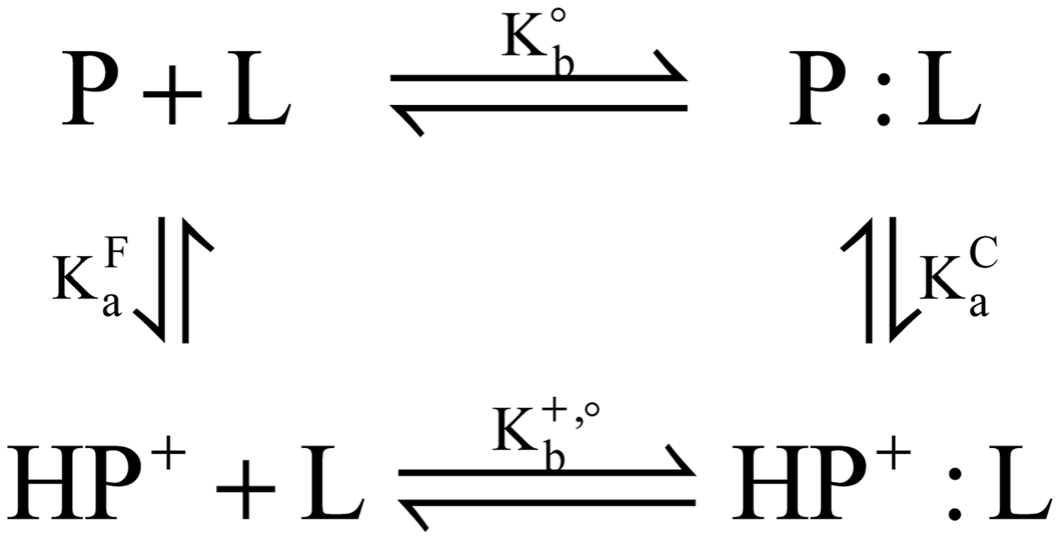
Thermodynamic cycle for complex formation between a protein (P) with a single titratable site and a ligand (L).

First, consider complex formation between a protein (P) with a single titratable site and a ligand (L) that does not titrate in the biological range of pH levels. The association can be expressed as a general equation with the apparent equilibrium constant, K_app_:

$$\{\text{P}\}+\text{L}\overset{{\text{K}}_{\text{app}}}{⇄}\{\text{PL}\},$$ (4)

where the curly braces indicate that the ensembles of protein and protein-ligand complex (PL) may contain different protonated forms of the titratable species. K_app_ can be expressed in terms of binding polynomials through an application of the thermodynamic cycle for proton-linked ligand binding shown in Fig 10:

$${\text{K}}_{\text{app}}=\frac{\left[\text{PL}]+[{\text{HPL}}^{+}\right]}{\left(\left[\text{P}]+[{\text{HP}}^{+}\right]\right)[\text{L}]}={\text{K}}_{\text{b}}^{\circ }\frac{\left(1+\frac{\left[{\text{HPL}}^{+}\right]}{\left[\text{PL}\right]}\right)}{\left(1+\frac{\left[{\text{HP}}^{+}\right]}{\left[\text{P}\right]}\right)},$$ (5)

where the concentrations of the given species, instead of activities, are shown assuming ideal dilute solutions and K°_b_ is the equilibrium constant of binding for a reference reaction in which net proton transfer is ignored.

The overall free energy of binding (ΔG°) can then be expressed by using logarithmic representations of the acid dissociation constants for the free protein (pK_a_^F^) and protein-ligand complex (pK_a_^C^):

$$\Delta {\text{G}}^{\circ }(\text{pH})=\Delta {\text{G}}_{\text{ref}}^{\circ }-{\text{k}}_{\text{B}}\text{Tln}\left(\frac{1+10^{{\text{pK}}_{\text{a}}^{\text{C}}-\text{pH}}}{1+10^{{\text{pK}}_{\text{a}}^{\text{F}}-\text{pH}}}\right),$$ (6)

where ΔG°_ref_ is the free energy of binding for the reference reaction.

In cases where proton binding to different titratable sites may be cooperative, Wyman [111] derived a relation between K_app_ and pH such that

$$\frac{\partial {\text{lnK}}_{\text{app}}}{\partial \text{ln}[{\text{H}}^{+}]}=\Delta {\text{ν}}_{{\text{H}}^{+}}={\text{Z}}_{\text{PL}}-({\text{Z}}_{\text{P}}+{\text{Z}}_{\text{L}}),$$ (7)

where, using the notation used by Tanford [91], Δν_H+_ is the change in the number of bound protons in the protein-ligand complex, relative to the number of protons bound to the protein and ligand individually. With ΔZ = Z_PL_−(Z_P_ + Z_L_), integration of Eq 7 provides a thermodynamic relation for proton-linked ligand binding where titratable sites may interact:

$$\Delta {\text{G}}^{\circ }(\text{pH})=\Delta {\text{G}}_{\text{ref},\text{pH}}^{\circ }-{\text{k}}_{\text{B}}\text{Tln}(10)\int_{{\text{pH}}_{\text{ref}}}^{\text{pH}}\left\{{\text{Z}}_{\text{PL}}(\text{pH})-{\text{Z}}_{\text{P}}(\text{pH})-{\text{Z}}_{\text{L}}(\text{pH})\right\}\text{dpH},$$ (8)

where Z_PL_, Z_P_, and Z_L_ are the total charges for protein-ligand complex, protein, and ligand, respectively, obtained from the pH-REMD simulations. Eq 8 provides framework for computing the pH-dependent binding free energy through a correction term to the reference free energy of binding. In this work, Z_L_ is omitted from consideration since the inhibitors considered here do not titrate in the physiological pH range.

### Preparation of the BACE-1 Systems for Simulations

The X-ray crystallographic structures of BACE-1 in complex with inhibitors N-[(1S,1R)-benzyl-3-(cyclopropylamino)-2-hydroxypropyl]-5-[methyl(methylsulfonyl)amino-N’-[(1R)-1-phenylethyl]isophthalamide (PDB ID 2B8L) [112]; N-[(1S,2S,4R)-2-hydroxy-1-isobutyl-5-({(1S)-1-[(isopropylamino)carbonyl]-2-methylpropyl}amino-4-methyl-5-oxopentyl]-5-methyl(methylsulfonyl)amino]-N’-[(1R)-1-phenylethyl]isophthalamide (PDB ID 2P4J) [113]; N~2~-[(2R,4S,5S)-5-{[N-{[3,5-dimethyl-1H-pyrazol-1-yl)methoxy]carbonyl}-3-(methylsulfonyl)-_L_-alanyl]amino}-4-hydroxy-2,7-dimethyloctanoyl]-N-isobutyl-_L_-valinamide (PDB ID 2G94) [100]; 3,{5-[(1R)-1-amino-1-methyl-2-phenylethyl]-1,3,4-oxadiazol-2-yl}-N-[(1R)-1-(4-fluorophenyl)ethyl]-5-[methyl(methylsulfonyl)amino]benzamide (PDB ID 2IRZ) [114]; and N1-((2S,3S,5R)-3-amino-6-(4-fluorophenylamino)-5-methyl-6-oxo-1-phenylhexan-2-yl)-N3,N3-dipropylisophthalamide (PDB ID 2FDP) [102] were used to study the pH-dependent inhibitor binding to BACE-1. The chemical structures of the inhibitors are shown in Fig 2. A segment between residues Gly158 and Ser169 is not solved in 2B8L, 2IRZ, and 2FDP crystal structures and hence this loop was built by homology modeling using the Structure Prediction Wizard module of Schrödinger’s Prime program [115–117]. The FASTA sequence of protein including the missing loop region for each X-ray structure was obtained from UniProt [118]. The mutation AWA that exists in 2B8L and 2IRZ structures made for crystallography were corrected to the original sequence of KWE [112,114]. Using the homologs found by BLAST search algorithm [119], a chimera model containing the missing loop region was built for each structure. The homology-modeled loop region was energy-refined for relaxation using the Refine Loops panel of the Prime program [115]. Apo structure of BACE-1 was generated by removing the bound inhibitor from the refined 2B8L structure to even out any effects that may arise from homology modeling.

The geometries of the inhibitors were optimized at the B3LYP/6-31G(d) level of theory [120–123] using the Gaussian 09 suite of programs [124]. The electronic potentials (ESP) for the optimized geometries of the inhibitors were computed using MK radii [125] at the HF/6-31G(d) level of theory. Subsequently, the atomic point charges were computed from the ESPs by applying the RESP procedure using the antechamber module [126] in the AmberTools 14 suite of programs [95]. All other force field terms including Lennard-Jones parameters for use in molecular dynamics (MD) simulations were taken from the general AMBER force field (GAFF) [127].

### Conventional Molecular Dynamics Simulations

Prior to the pH-REMD simulations, each system was subject to short conventional molecular dynamics (cMD) simulations for equilibration purposes. Protonation states of the titratable groups are assigned using the PROPKA web server [75,92–94]. All protein force field parameters are taken from the AMBER ff14SB force field [95], while the ligand parameters are taken from the AMBER GAFF force field [127]. Each system was solvated with TIP3P water [128] and counterions were added to neutralize the system by tleap program [129]. Water molecules were first minimized and simulated for 150 ps in the NPT ensemble with a harmonic restraint of 2.0 kcal/mol Å^2^ on the protein and ligand heavy atoms to relax the water. The entire system was then minimized and heated to 300 K over 500 ps. Two equilibrations with respective duration of 200 ps were performed. First, the system was equilibrated at constant volume and temperature (NVT) using a Langevin thermostat [130]. Following this, the second equilibration was carried out at constant pressure and temperature (NPT) using a Berendsen barostat (ntp = 1) [131] with isotropic position scaling to bring the system to a stable density. A 100 ns cMD production was then performed in the NVT ensemble. The Particle Mesh Ewald summation method was used to compute long-range electrostatic interactions [132,133], and short-range non-bonded interactions are truncated at 8 Å in the periodic boundary conditions. All dynamics are conducted using the *pmemd*.*cuda* module of AMBER 14 suite of programs [95,130,134]. The RMSD of the apo structure indicated a convergence to the starting conformation after first 20 ns, ensuring the stability of the system (S10 Fig).

### Constant pH Replica Exchange Molecular Dynamics Simulations Details

Preliminary investigation of the pK_a_ shift of the titratable groups of BACE-1 upon inhibitor binding was carried out using the PROPKA web server by submitting the apo and holo structures [75,92–94]. The results indicated binding-induced pK_a_ shifts for a number of ionizable residues, with the most pronounced shift for the aspartyl dyad. A total of ten ionizable residues within 12 Å of the active site were chosen for titration, Asp32, Asp106, Asp138, Asp223, Asp228, Glu116, Glu265, Glu339, His45, and Tyr71. As the titratable groups in the inhibitors considered in this study have pK_a_’s above 12, the titration was carried out on the chosen protein side chains only.

pH-REMD simulations were performed using the *pmemd*.*cuda*.*MPI* module of AMBER 14 suite of programs for the pH range between 1 and 12 stepped by 1 pH unit [95,108]. All simulations employed the generalized Born (GB)-Neck 2 implicit solvent model (igb = 8) [105] with a salt concentration of 0.1 M. To ensure equilibration in the implicit solvent, a 5,000 step minimization was carried out for each system starting from the conformation obtained from the cMD simulations, with positional restraints on all heavy atoms with a force constant of 20 kcal/mol Å^2^. The system was then heated to 300 K over 500 ps using a Langevin thermostat while maintaining the positional restraints applied to all heavy atoms with a force constant of 5 kcal/mol Å^2^, followed by a 1 ns equilibration. The Monte Carlo (MC) moves for titration were performed during the production stage only, where the MC steps taken every 10 fs and exchange between replicas at adjacent pH attempted every 100 steps, *i*.*e*., 200 fs with a 2 fs time step. The production simulations were carried out for 60 ns and data from last 50 ns were used for analyses. In the equilibration and production steps, the bonds involving hydrogen atoms were constrained using the SHAKE algorithm [135].

### Simulation Analyses

RMSF and clustering analyses, and reconstruction of the pH-based trajectories from the pH-REMD simulations were performed using cpptraj program in the AmberTools 14 suite of programs [95]. Clustering analyses for the pH-based trajectories used pairwise RMSDs computed for Cα atoms between frames to divide the trajectories into five clusters using the average-linkage algorithm [136]. Fitting of titration data to the Hill equation (Eq 3) to obtain the pK_a_ values was carried out using Matlab [137].

## Supporting Information

S1 Fig Change in distance between the dyad and flap region observed in the cMD simulations. (A) 2P4J. (B) 2G94. (C) 2IRZ.
(TIF)

S2 Fig Distribution of distances between the dyad and flap at acidic (pH 1 to 3; red) and basic (pH 9 to 11; blue) pH. (A) 2P4J. (B) 2G94. (C) 2IRZ.
(TIF)

S3 Fig Titration curves from the pH-REMD simulations of apo BACE-1. (TIF)

S4 Fig Titration curves from the pH-REMD simulations of the 2P4J system, shown in purple. Respective titration curves of the dyad in apo BACE-1 are shown in green.
(TIF)

S5 Fig Titration curves from the pH-REMD simulations of the 2G94 system, shown in purple. Respective titration curves of the dyad in apo BACE-1 are shown in green.
(TIF)

S6 Fig Titration curves from the pH-REMD simulations of the 2IRZ system, shown in purple. Respective titration curves of the dyad in apo BACE-1 are shown in green.
(TIF)

S7 Fig Titration curves from the pH-REMD simulations of the 2B8L system, shown in purple. Respective titration curves of the dyad in apo BACE-1 are shown in green.
(TIF)

S8 Fig Titration curves from the pH-REMD simulations of the 2FDP system, shown in purple. Respective titration curves of the dyad in apo BACE-1 are shown in green.
(TIF)

S9 Fig Binding free energy profiles of inhibitors as functions of pH. (A) 2P4J. (B) 2G94. (C) 2IRZ.
(TIF)

S10 Fig RMSD of apo BACE-1 in the cMD simulation. (TIF)
