# Supplementary figures and images for: Conformational Dynamics and Binding Free Energies of Inhibitors of BACE-1: From the Perspective of Protonation Equilibria
Source: PLoS Comput Biol. 2015 Oct 27;11(10):e1004341. doi: 10.1371/journal.pcbi.1004341 (PMC4623973; doi:10.1371/journal.pcbi.1004341)

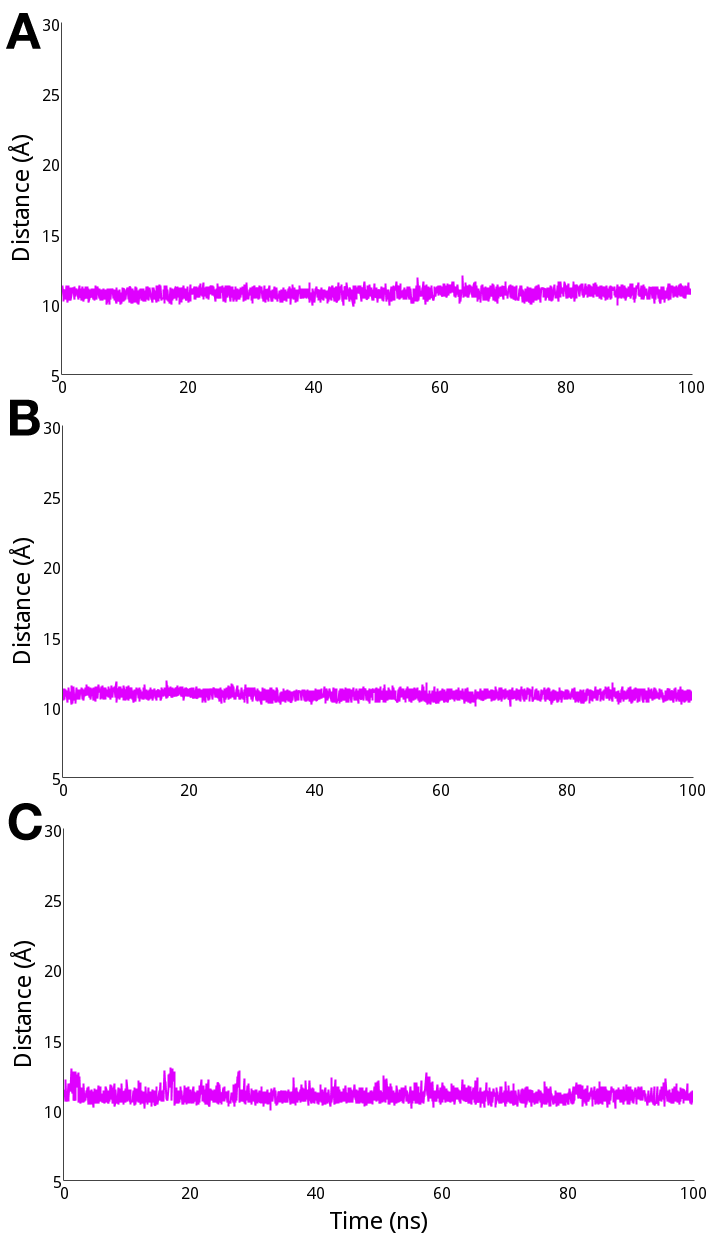

Supplement: S1 Fig — (A) 2P4J. (B) 2G94. (C) 2IRZ. (TIF) [file pcbi.1004341.s001.tif]

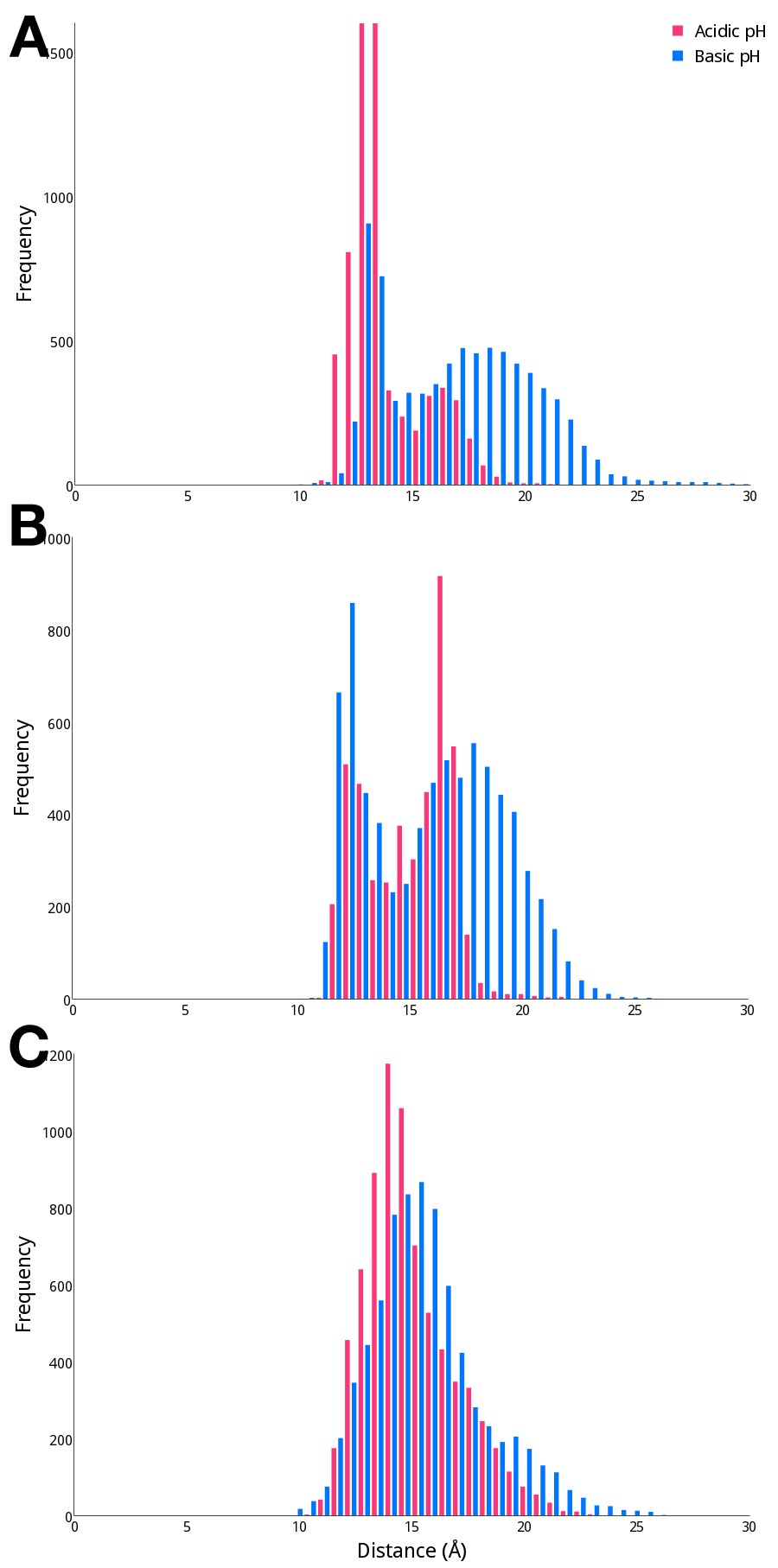

Supplement: S2 Fig — (B) 2G94. (C) 2IRZ. (TIF) [file pcbi.1004341.s002.tif]

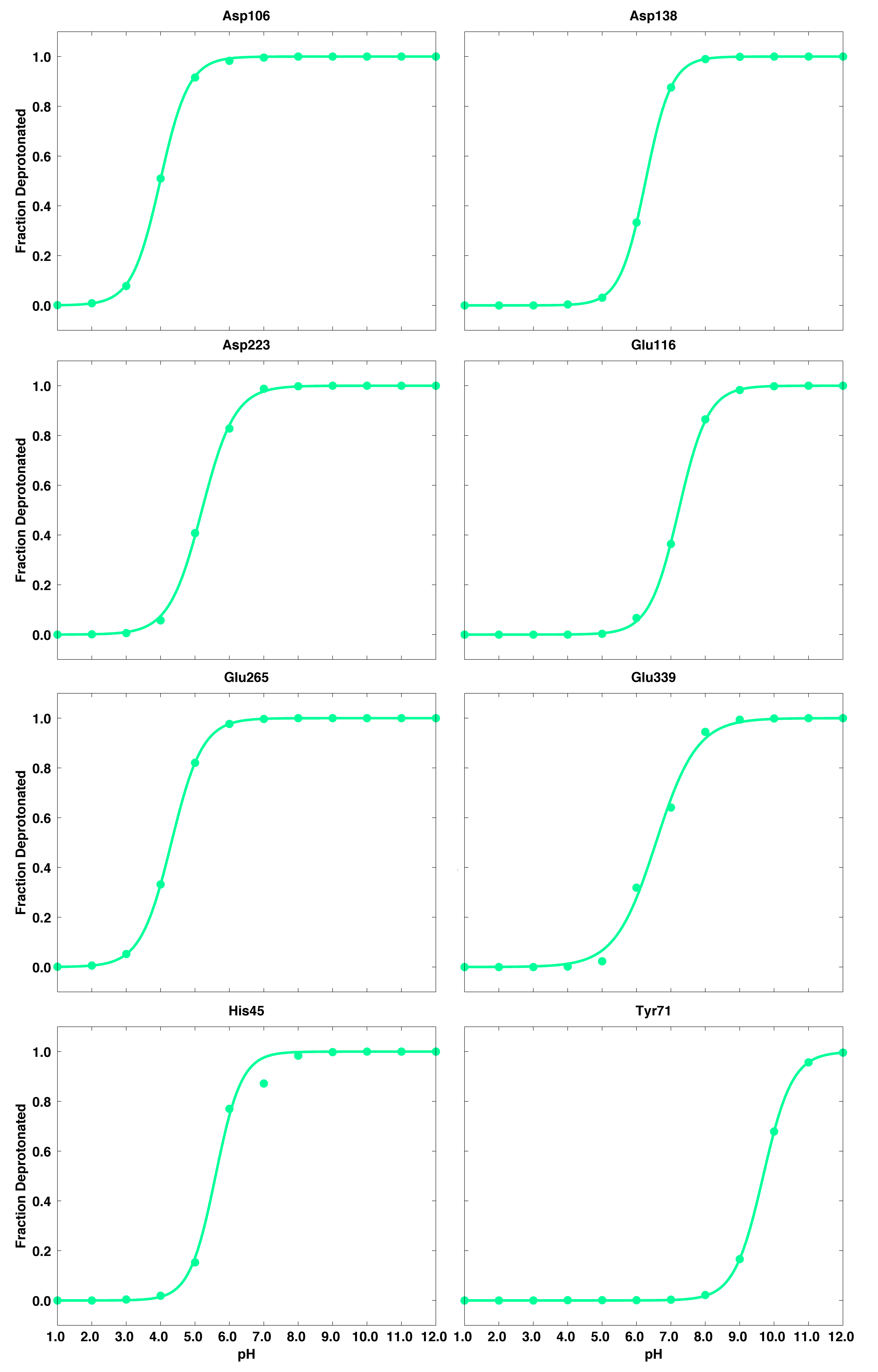

Supplement: S3 Fig — (TIF) [file pcbi.1004341.s003.tif]

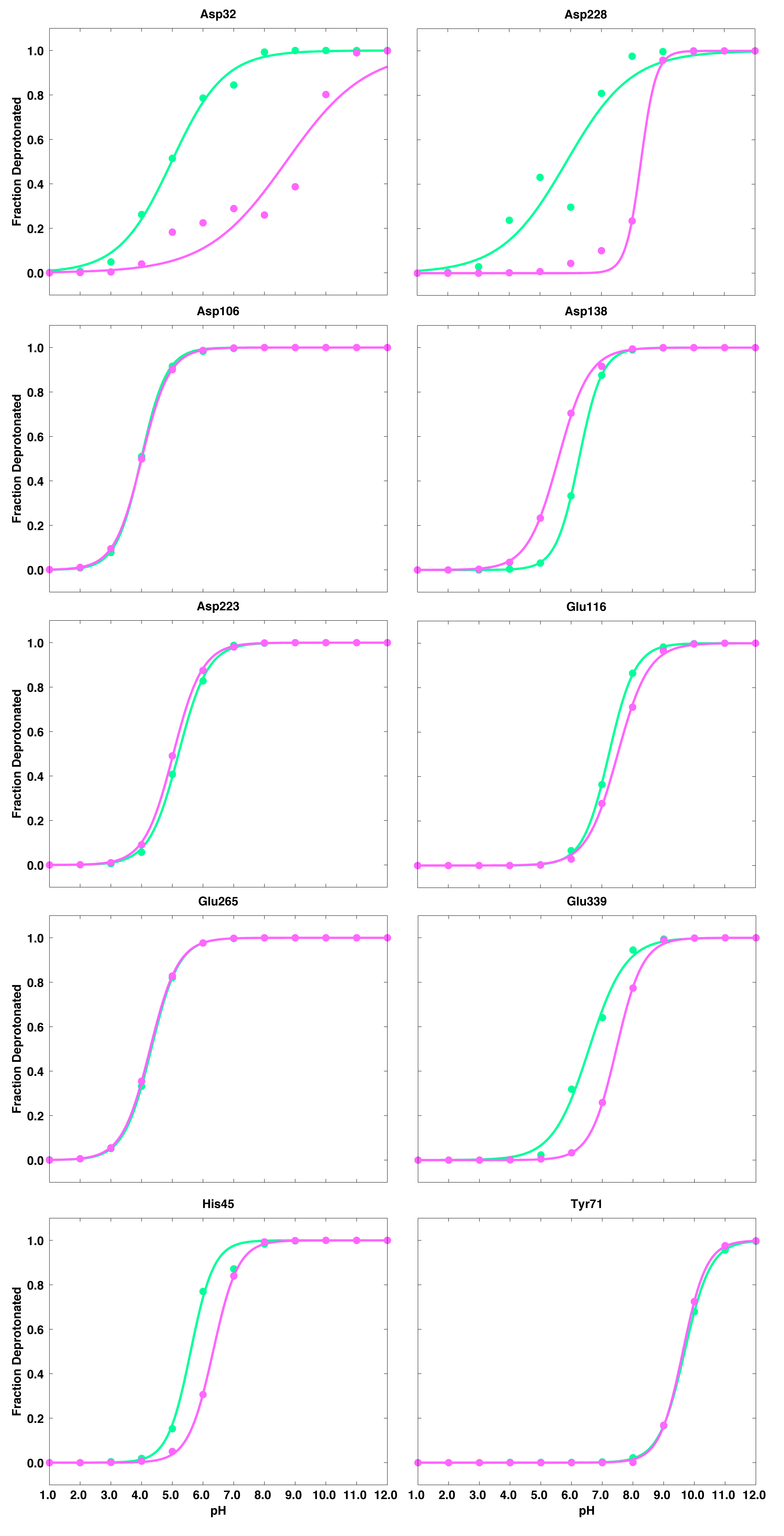

Supplement: S4 Fig — Respective titration curves of the dyad in apo BACE-1 are shown in green. (TIF) [file pcbi.1004341.s004.tif]

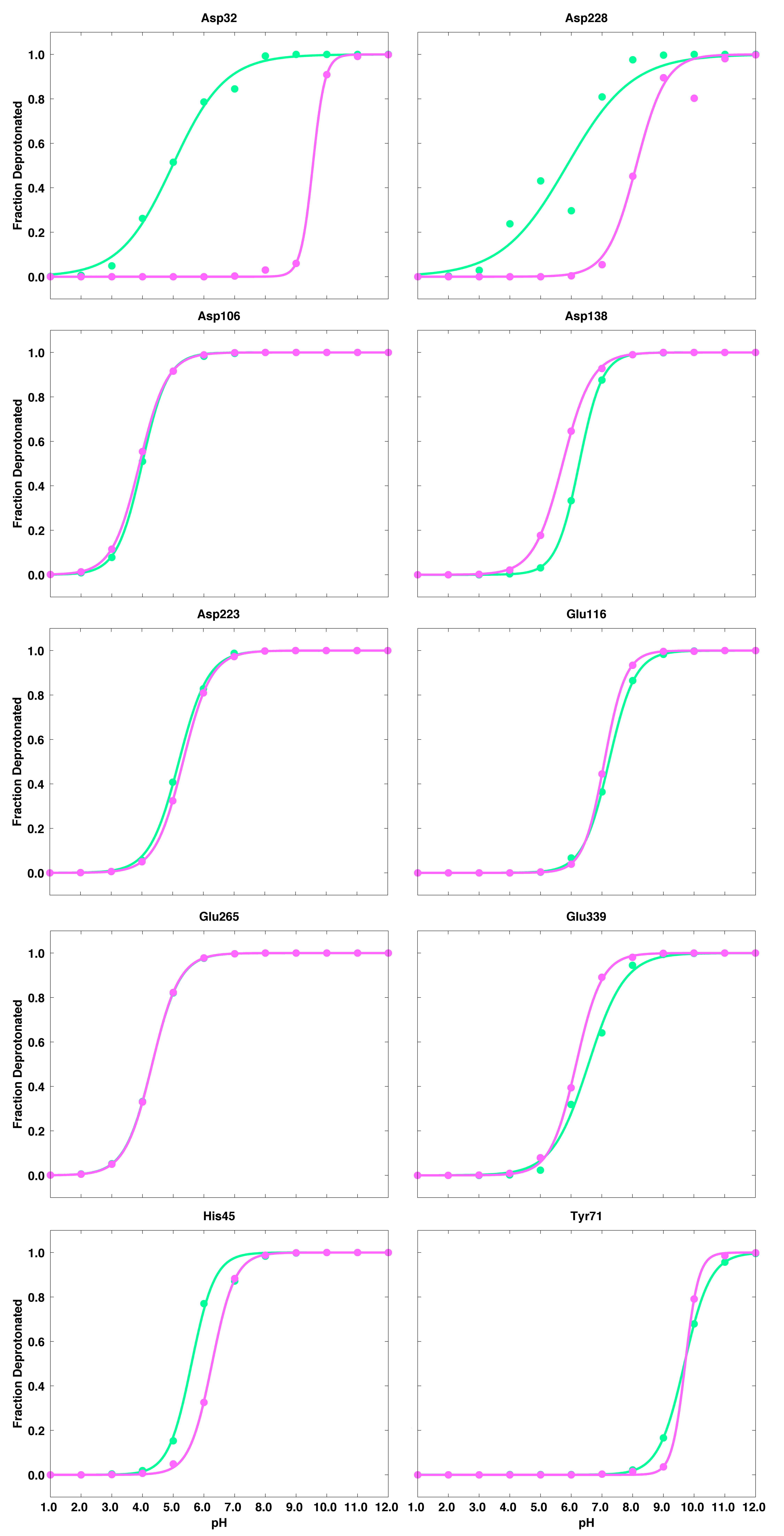

Supplement: S5 Fig — Respective titration curves of the dyad in apo BACE-1 are shown in green. (TIF) [file pcbi.1004341.s005.tif]

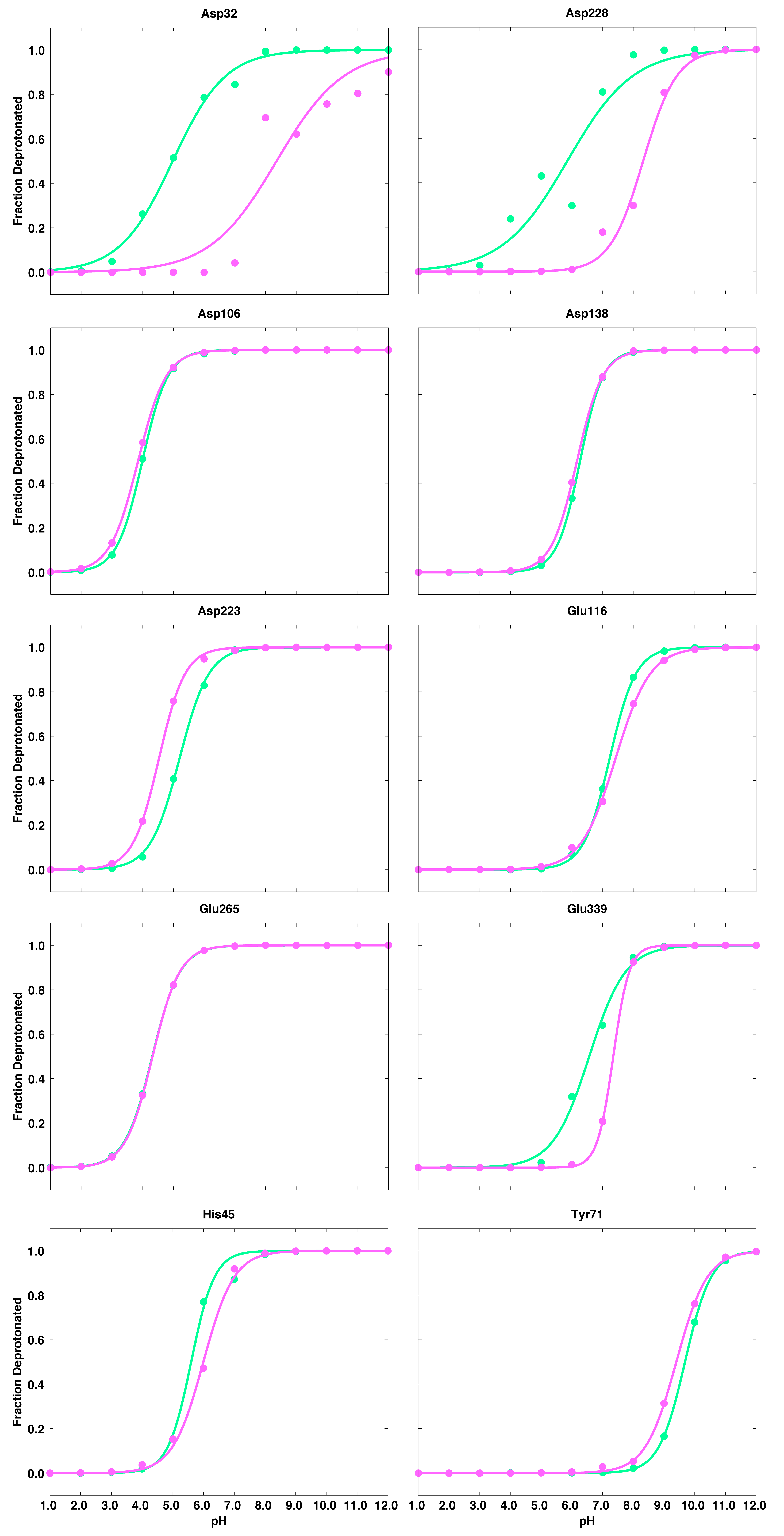

Supplement: S6 Fig — Respective titration curves of the dyad in apo BACE-1 are shown in green. (TIF) [file pcbi.1004341.s006.tif]

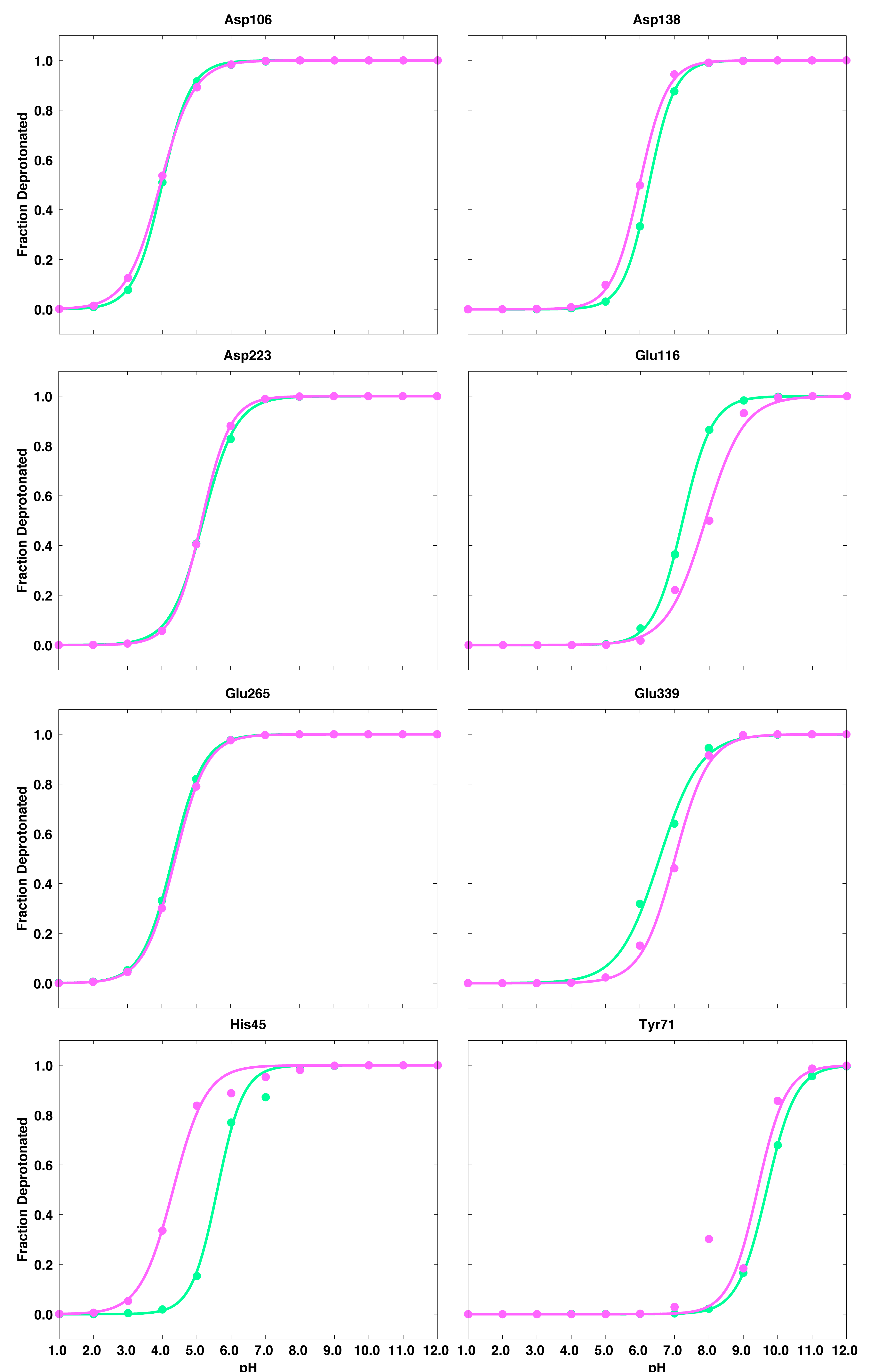

Supplement: S7 Fig — Respective titration curves of the dyad in apo BACE-1 are shown in green. (TIF) [file pcbi.1004341.s007.tif]

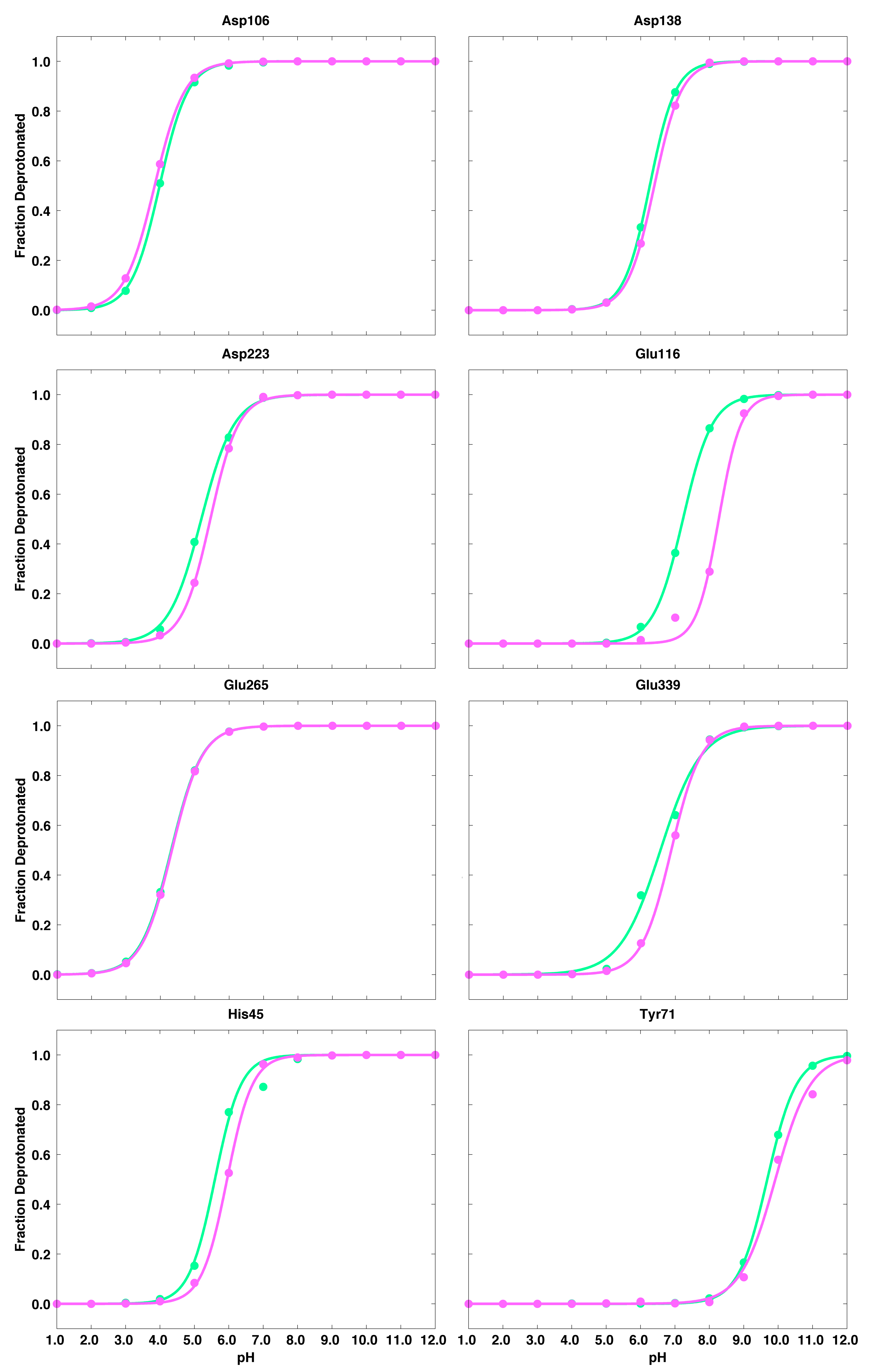

Supplement: S8 Fig — Respective titration curves of the dyad in apo BACE-1 are shown in green. (TIF) [file pcbi.1004341.s008.tif]

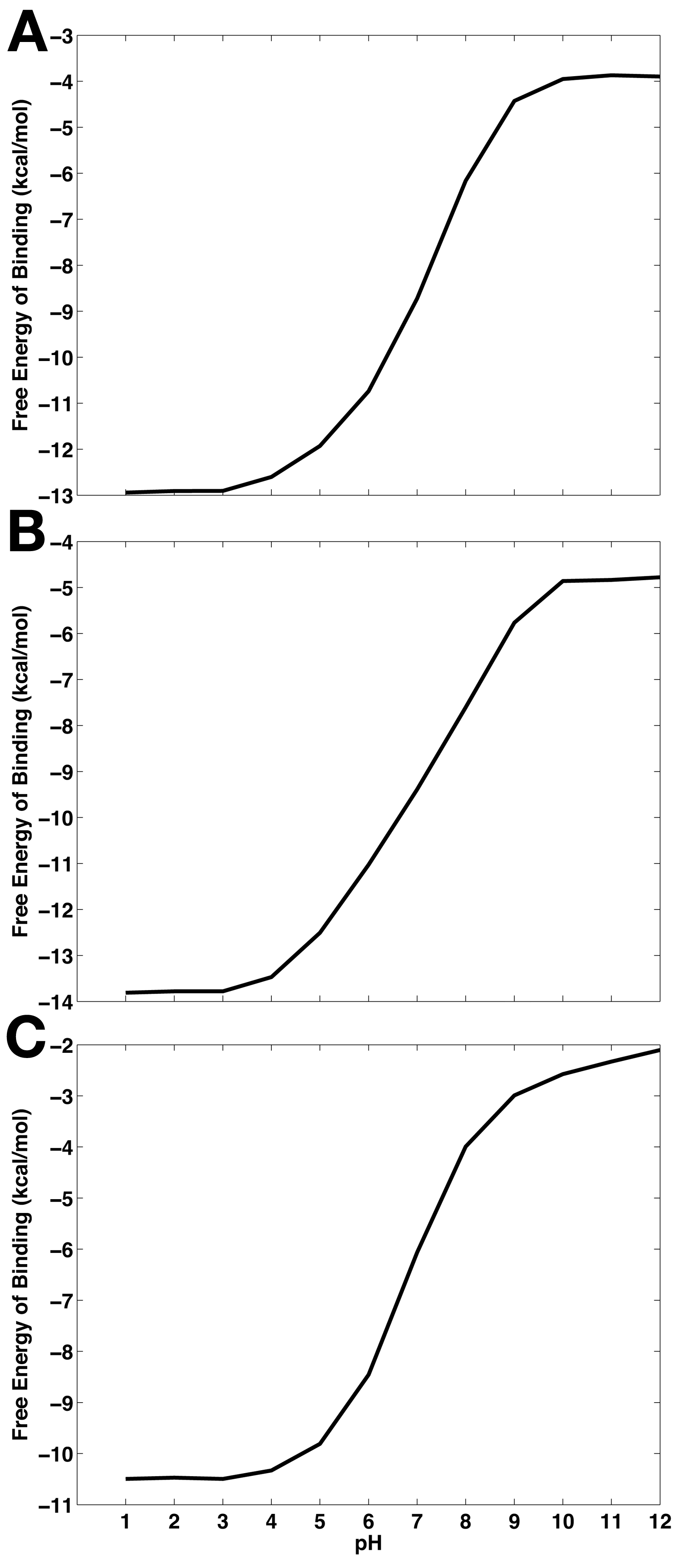

Supplement: S9 Fig — (A) 2P4J. (B) 2G94. (C) 2IRZ. (TIF) [file pcbi.1004341.s009.tif]

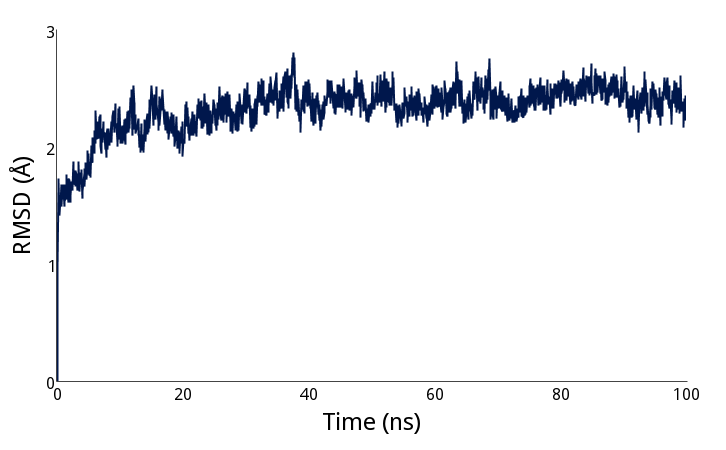

Supplement: S10 Fig — (TIF) [file pcbi.1004341.s010.tif]
